# Transcriptional profiling of liver during the critical embryo-to-hatchling transition period in the chicken (*Gallus gallus*)

**DOI:** 10.1186/s12864-018-5080-4

**Published:** 2018-09-21

**Authors:** Larry A. Cogburn, Nares Trakooljul, Chuming Chen, Hongzhan Huang, Cathy H. Wu, Wilfrid Carré, Xiaofei Wang, Harold B. White

**Affiliations:** 1 Department of Animal and Food Sciences, University of Delaware, Newark, DE 19716 USA; 2 Center for Bioinformatics and Computational Biology, University of Delaware, Newark, DE 19716 USA; 3 Department of Chemistry and Biochemistry, University of Delaware, Newark, DE 19716 USA; 4 Present Address: Leibniz Institute for Farm Animal Biology (FBN), Institute for Genome Biology, 18196 Dummerstorf, Germany; 5 Laboratoire de Génétique Moléculaire et Génomique, CHU Pontchaillou, 35033 Rennes, France; 6 Department of Biological Sciences, Tennessee State University, Nashville, TN 37209 USA

**Keywords:** Metabolic switch, Lipolysis, Lipogenesis, Opposing up-stream regulators, THRSPA, SERTAD2, Kruppel-like factors, Hepatic FKER, Coagulation system, Homeorhetric regulation of metabolism, Transcriptional activation/inactivation

## Abstract

**Background:**

Although hatching is perhaps the most abrupt and profound metabolic challenge that a chicken must undergo; there have been no attempts to functionally map the metabolic pathways induced in liver during the embryo-to-hatchling transition. Furthermore, we know very little about the metabolic and regulatory factors that regulate lipid metabolism in late embryos or newly-hatched chicks. In the present study, we examined hepatic transcriptomes of 12 embryos and 12 hatchling chicks during the peri-hatch period—or the metabolic switch from chorioallantoic to pulmonary respiration.

**Results:**

Initial hierarchical clustering revealed two distinct, albeit opposing, patterns of hepatic gene expression. Cluster A genes are largely lipolytic and highly expressed in embryos. While, Cluster B genes are lipogenic/thermogenic and mainly controlled by the lipogenic transcription factor *THRSPA*. Using pairwise comparisons of embryo and hatchling ages, we found 1272 genes that were differentially expressed between embryos and hatchling chicks, including 24 transcription factors and 284 genes that regulate lipid metabolism. The three most differentially-expressed transcripts found in liver of embryos were *MOGAT1, DIO3* and *PDK4*, whereas *THRSPA, FASN* and *DIO2* were highest in hatchlings. An unusual finding was the “ectopic” and extremely high differentially expression of seven feather keratin transcripts in liver of 16 day embryos, which coincides with engorgement of liver with yolk lipids. Gene interaction networks show several transcription factors, transcriptional co-activators/co-inhibitors and their downstream genes that exert a ‘ying-yang’ action on lipid metabolism during the embryo-to-hatching transition. These upstream regulators include ligand-activated transcription factors, sirtuins and Kruppel-like factors.

**Conclusions:**

Our genome-wide transcriptional analysis has greatly expanded the hepatic repertoire of regulatory and metabolic genes involved in the embryo-to-hatchling transition. New knowledge was gained on interactive transcriptional networks and metabolic pathways that enable the abrupt switch from *ectothermy* (embryo) to *endothermy* (hatchling) in the chicken. Several transcription factors and their coactivators/co-inhibitors appear to exert opposing actions on lipid metabolism, leading to the predominance of lipolysis in embryos and lipogenesis in hatchlings. Our analysis of hepatic transcriptomes has enabled discovery of opposing, interconnected and interdependent transcriptional regulators that provide precise *ying-yang* or *homeorhetic* regulation of lipid metabolism during the critical embryo-to-hatchling transition.

**Electronic supplementary material:**

The online version of this article (10.1186/s12864-018-5080-4) contains supplementary material, which is available to authorized users.

## Background

The developing mammalian embryo is completely dependent upon its mother and her placenta to supply nutrients, exchange respiratory gases, and to remove nitrogenous waste products. Quite the opposite is true among amniotes (i.e., birds and reptiles), which must undergo independent embryonic development in a pre-formed and completely enclosed system—the cleidoic egg. In chickens, the principal source of energy for late embryonic growth is derived from yolk lipids stored in the egg by the hen. The embryo exhibits exponential growth between days 13 to 18 of embryonic development (E13 to E18), when the total energy required for growth comes from β-oxidation of fatty acids derived from yolk lipids [1, 2]. The final phase of embryonic development is marked by a dramatic accumulation of cholesterol esters in the liver (> 30% of its dry mass) [3]. Just prior to hatching, the yolk sac and its remaining lipids are retracted into the embryo’s visceral cavity to fuel the ensuing metabolic switch to endothermy in hatchling chicks. Yolk lipids are utilized by the newly hatched chicken from direct transfer into circulation via transport from the yolk stalk into the small intestine [4]. Although fatty acids are readily absorbed from the lipid drenched intestine, the absorption of carbohydrates and amino acids from ingested high-energy feed is delayed until expression of intestinal enzymes and cotransporters (glucose/sodium/amino acid) reach adequate levels [4–7]. A recent transcriptional analysis, using serial analysis of gene expression (SAGE), of the chick embryo yolk sac between E13 and E21 [8] provides the first view of genome-wide transcriptional changes that occur during transport of lipid from the yolk sac into the liver prior to hatching. The high abundance of yolk lipids and their transporters (i.e., lipoproteins) appear to interfere with absorption and utilization of nutrients from the intestine of the immediate post-hatch chick [4–6]. Thus, the newly hatched chick must undergo a dramatic metabolic shift from ectothermy, with an exclusive dependence on yolk lipids, to endothermy which depends on utilization of ingested feed—mainly carbohydrates and protein [5]. Early hepatic expression of several lipogenic enzymes [9–11] provides the chick with the ability to convert dietary carbohydrate into fat stores [2]. This sudden switch from a dependence on fat stored in yolk (embryos) to utilization of nutrients in feed (hatchling) requires major shifts in nutrient transport and metabolism that are controlled by yet uncharted genetic pathways. Although newly hatched chicks can survive for several days on residual yolk, delayed feeding prevents full recovery and normal growth, despite a brief compensatory growth spurt [12]. Prolonged starvation during the immediate post-hatch period retards body and muscle growth, which is irreversible due to cellular changes in skeletal muscle [13]. Although hatching is perhaps the most abrupt and profound metabolic challenge that a chicken must experience, there have been no attempts to functionally map the metabolic pathways induced by nutrients in the chick’s first meal. Furthermore, we know very little about transcriptional control of multiple metabolic and regulatory factors that control metabolism during the critical immediate post-hatch period.

The chicken embryo develops as an aquatic ectotherm with respiratory gas exchange via the chorioallantoic membrane. On the last day of embryonation (E21), the fully-developed embryo must suddenly convert to pulmonary respiration after pipping through the eggshell. During the peri-hatch period, the precocious chick becomes an endotherm with a very high and self-sustaining metabolic rate maintained by a fully-functional thermoregulatory system. The thyroid axis plays a major role in regulating metabolism and energy expenditure. Hence, the activity and importance of the thyroid axis has been extensively studied during the peri-hatch period of the chicken [14–18]. The thyroid axis prepares the embryo for this metabolic jump with decreased activity of the inactivating deiodinase (*DIO3*) at E20, whereas the activity of the activating deiodinases (*DIO1* and *DIO2*) increases in E20 embryos during hatching and afterwards in the hatchling. The pro-thyroid hormone (T_4_) is readily deactivated to reverse rT_3_ by *DIO3* in liver of E16 and E18 embryos; then with onset of pulmonary respiration, the activity of (DIO1) and (DIO2) increases in liver to meet the ensuing energy demands of hatching and endothermy. The embryo must switch from chorio-allantoic to pulmonary respiration and instantly meet the high metabolic demands of an endotherm—the hatchling chick. Therefore, it is essential to understand the transcriptional networks that integrate and control function of the endocrine system to meet the *dynamic* metabolic demands of growth imposed upon the liver.

Our first glimpse of the liver transcriptome during the chicken’s peri-hatch period came from an earlier low-resolution transcriptional scan using the original chicken liver (3.2 K) microarray [19]. This initial transcriptional study using our prototype microarray was completed with 24 liver samples taken during the peri-hatch period [embryonic day 16 (E16) to post-hatch day 9 (D9)]. Self-organizing maps (SOMs) clustering of the 3.2 K microarray data identified two distinct gene expression profiles in liver. Cluster A contained differentially-expressed genes (DEGs) with very high expression in hatching liver, while Cluster B DEGs showed higher expression in embryo liver (*see* Fig. 2 in [19]). Hepatic expression of several lipolytic genes was higher in embryos and then abruptly decreased after hatching. In contrast, many up-regulated genes in liver of hatchling chicks control lipogenesis and energy metabolism (*THRSPΑ*, *SCD*, *FASN*, *ME1*, *HMGCS*, and *EVOL6*)*.*

The present high-resolution genome-wide transcriptional analysis of the liver during the peri-hatch period has greatly expanded our hepatic repertoire of regulatory and metabolic genes involved in the embryo-to-hatchling transition. Several transcription factors were identified that appear to exert opposing actions, which lead to the predominance of lipolysis in embryos or lipogenesis in hatchlings. Furthermore, we have gained new knowledge on interactive transcriptional networks and metabolic pathways that enable the abrupt switch from *ectothermy* (embryo) to *endothermy* (hatchling) in the chicken.

## Methods

### Animal care and use

Fertile eggs from Ross x Ross broiler (meat-type) chickens were purchased from a commercial hatchery (Moyer’s, Quakertown, PA). The eggs were incubated in an automated incubator (Jamesway Incubator Co., Ontario CAN) at 39 °C and 95% relative humidity. At E20, eggs containing viable embryos were transferred to hatching trays to determine the approximate time of hatch (i.e., wetness of down). Chicks hatched within a 4 h period on the 21st day of incubation were wing-banded and held in the incubator for an additional 4 h. The remaining one day-old (D1) chicks were vaccinated against Marek’s disease virus and transferred to a heated battery brooder (Petersime Incubator Co., Gettysburg OH) maintained at 33 °C. The chicks received a commercial starter ration (22% crude protein and 3100 kcal ME/kg) and water ad libitum throughout the experiment. After continuous light for the first two days, the birds were maintained on a 20 h light: 4 h dark (20 L:4D) photoperiod. All protocols used in this study were in accordance with the United States Department of Agriculture guidelines on the use of agricultural animals in research and approved by the University of Delaware Animal Care and Use Committee.

### Experimental design of hepatic transcriptional profiling study

The experimental design used for the present transcriptional profiling with Affymetrix Chicken Genome arrays was similar to the design utilized in our original 3.2 K microarray analysis of liver in peri-hatch chickens [19]. Four male embryos were killed for collection of liver samples on embryonic day 16 (E16), E18 and E20 and four male hatchlings taken at 1, 3 and 9 day (D) of age. The D1 liver samples were taken from hatched chicks held in the hatching incubator (38 °C) for 4 h. After visual verification of male sex (presence of testes), each liver was quickly removed, placed into a 15 ml Nalgene cryogenic vial and snap frozen in liquid nitrogen. The remaining chicks were provide with feed and water ad libitum and housed in a heated battery brooder maintained at under a 20 L:4D photoperiod. The frozen liver samples were transferred to an ultra-low freezer (− 85 °C) and stored until isolation of total RNA.

### Extraction and purification of total RNA

Total RNA was purified from each liver sample using an RNeasy Mini kit with on column DNase I treatment, as recommended by the manufacturer’s (Qiagen, Valencia, CA). Quantitation of total RNA was done using a NanoDrop spectrophotometer (NanoDrop, Wilmington DE) and the RNA was diluted to a standard concentration (250 ng/μL). RNA quality was assessed using an Agilent 2100 Bioanalyzer and an Agilent RNA 6000 Nano Kit (Agilent Genomics, Santa Clara, CA). Liver RNA samples (1 μL each) were analyzed on a RNA 6000 Nano Chip. The ribosomal RNA ratio (28S/18S) was assessed for RNA integrity, where with a RNA Integrity Number (RIN) of 9 to 10 indicates RNA of high quality for use in amplification of RNA for the microarray analysis and for verification by qRT-PCR analysis. Each liver RNA sample was divided into two identical aliquots and stored frozen at − 85 °C until use in the transcriptional analyses below.

### RNA amplification, biotin labeling and hybridization of chicken genome arrays

Microarray-based transcriptome profiling was carried out using 24 Affymetrix GeneChip™ Chicken Genome Arrays and one-cycle target labeling protocol according to the manufacturer’s (Affymetrix) protocols. The experimental design for hybridization of the 24 chicken genome arrays was based on four replicate hybridization days, where all six ages were represented by one of four biological replicates (individual embryo or hatchling) per age group (Additional file 1). Briefly, 5 μg of total RNA was reverse-transcribed into first-strand cDNA using a T7-oligo(dT) promoter primers, followed by RNase H-mediated second-strand cDNA synthesis. and subsequently in vitro transcribed (IVT) into biotinylated complementary RNA (cRNA) in the present of T7 RNA Polymerase and biotinylated nucleotide analog/ribonucleotide mix using the Affymetrix IVT Labeling Kit (Affymetrix, Santa Clara, CA). The biotin-labeled cRNAs were cleaned up, fragmented and quality checked on an Agilent 2100 Bioanalyzer and hybridized to Chicken GeneChip™ Arrays for 16 h. The probe arrays were washed and stained on the Affymetrix GeneChip™ Fluidics Station and scanned for raw intensity data on the Agilent GeneChip™ Scanner using GeneChip™ Operating Software (GCOS) on the Affymetrix GeneChip™ Workstation. The raw cel files were then used for pre-processing and normalization as describe below.

### Processing, normalization and statistical analysis of genome array data

**Fig. 1:**
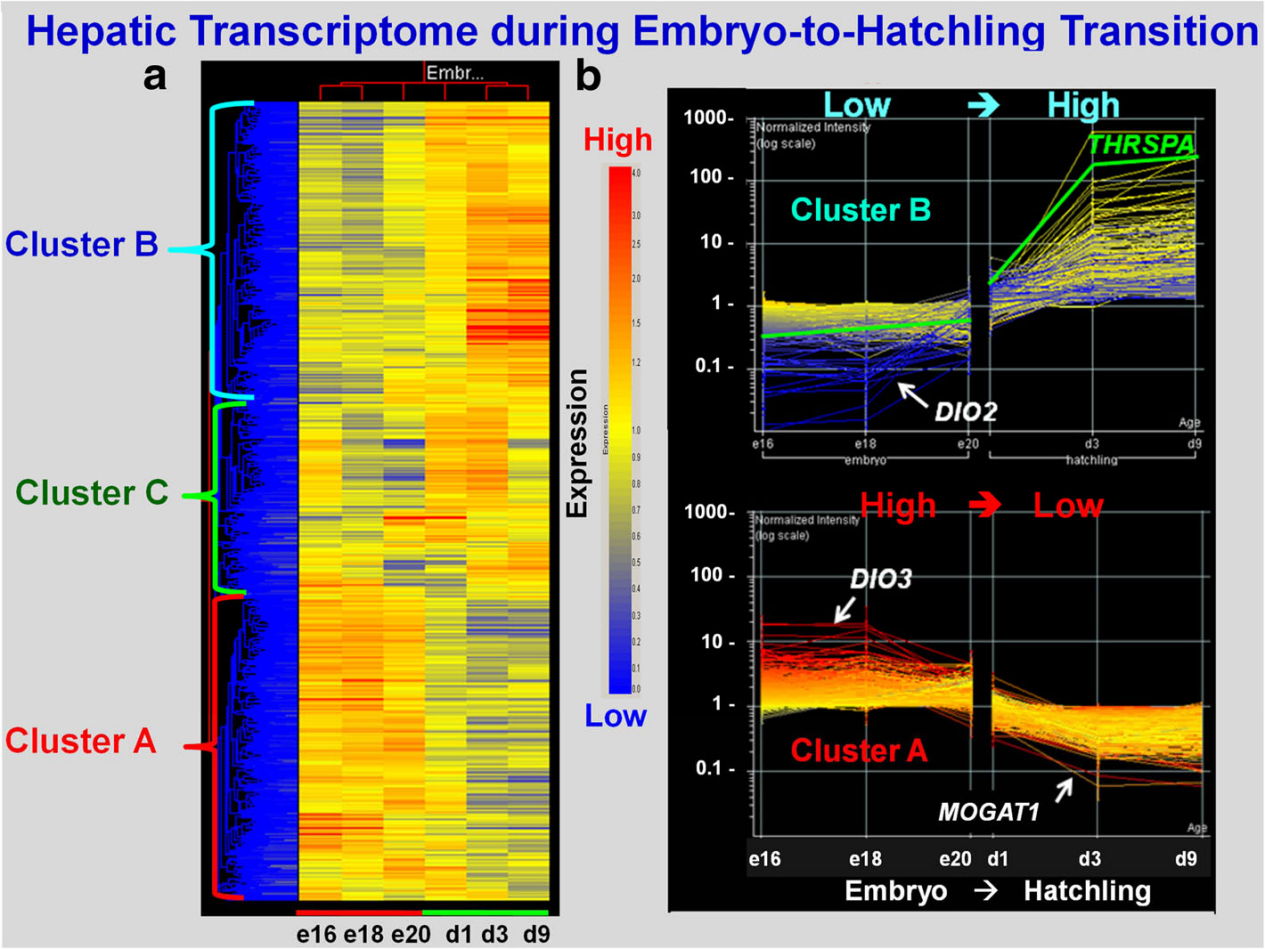
This figure provides an initial visual analysis of gene expression patterns found in liver of 12 embryos (E16, E18 and E20) and 12 hatchlings (D1, D3 and D9) during the critical embryo-to-hatchling transition. GeneSpring GX 7 software was only used for the preliminary analysis of differentially-expressed (FDR ≤ 0.01) genes (DEGs) and visualization of expression patterns from hierarchical (**a**) and k-means cluster (**b**) analyses. The heat map in Panel **a** clearly shows three distinct expression patterns of DEGs: Cluster A (high in embryos, low in hatchlings), Cluster B (low in embryos, high in hatchlings) and Cluster C (mixed pattern). K-means clustering (Panel **b)** provides higher resolution of DEGs found in Cluster A, including two examples: deiodinase 3 (*DIO3*) and monoacylclycerol O-acyltransferase 1 (MOGAT1), both have higher expression in embryos and lower abundance in hatchlings. In contrast, Cluster B contains DEGs with lower expression in embryos [i.e., deiodinase 2 (*DIO2*)] and much greater (log-scale) abundance in liver of hatchlings [i.e., thyroid hormone-responsive Spot 14 protein alpha (*THRSPA*)], especially in fully-fed D3 and D9 chicks

An initial visual analysis (including hierarchical and K-mean clustering) of the microarray data was completed using GeneSpring GX software (Agilent, Santa Clara, CA). Raw cell files from the Affymetrix GeneChip Chicken Genome Arrays were imported into GeneSpring software and unfiltered differentially-expressed genes (DEGs; FDR ≤ 0.01) identified for a preliminary hierarchical and k-means cluster analysis (Fig. 1 only).

Next, open-source R (version 3.3.3) [20] software packages, available from Bioconducter [21], were used for data processing and statistical analysis of the single-channel microarray data (raw cell files) obtained in this study. The software package affylmGUI (version 1.48.0) was used to perform normalization and to determine differential expression [22]. The robust multi-array average (RMA) method [23] was applied for background correction, quantile normalization and output of gene expression values on the log2 scale. Internally, affylmGUI uses the Linear Model Fit of LIMMA package (version 3.30.13) to assess differential expression of genes [24]. Statistical significance was adjusted for multiple testing by controlling the Benjamini and Hochberg’s false discovery rate [25], which was set at FDR ≤ 0.05. The Affymetrix chicken annotation data package [26] was then used to annotate the differentially-expressed genes (DEGs). LIMMA was used to make pairwise comparisons (contrasts) across embryo and hatchling ages and to filter DEGs by the FDR adjusted *p*-value (*P* ≤ 0.05) and a log2 ratio ± 0.75 cutoff. This gene expression level threshold of log2 ratio ± 0.75 (or a 1.68-fold difference) has proven useful for statistical analysis of microarray data in model plants [27].

### Ingenuity pathways analyses

An annotated list of DEGs for each pairwise contrast was then used as the input file for Ingenuity® Pathway Analysis (IPA), which provides functional annotation, mapping to canonical pathways and biological processes, and identification of gene interaction networks [28]. The DEGs accepted by IPA are considered as “Analysis Ready” (AR)-DEGs, if the gene is curated and annotated in the Ingenuity® Knowledge Base. However, the Ingenuity Knowledge Base is mainly based on mammalian biomedical literature and rather void of avian-specific genes. We used a combination of Human Genome Organisation (HUGO) gene symbol as the primary gene ID and the RefSeq Protein ID as the secondary gene ID to maximize the number of chicken DEGs accepted by IPA. The AR-DEGs were mapped onto canonical pathways, biological processes and gene interaction networks. IPA uses the Fisher’s Exact Test to indicate significance (*P* ≤ 0.05) of over-representation of AR-DE genes in canonical pathways and biological processes. The Ingenuity® Upstream Regulator Analysis was used to identify major transcription factors, their interaction with other upstream regulators, and their interactions with direct target genes to predict either activation or inhibition of upstream regulators. Lists of annotated AR-DEGs were generated by IPA and provided in Additional files.

### Verification of differential gene expression by quantitative RT-PCR analysis

A second aliquot of each total RNA sample from the 24 livers (above) was used for quantitative real-time PCR (qRT-PCR) analysis. The expression of 15 DEGs identified by microarray analysis was verified by qRT-PCR analysis (see Additional file 2). Selection of these candidate genes was based on their known function in pathways of interest or involvement in lipid metabolism. In additional, a panel of three invariant genes (*COX7A2L, PCF11* and *PRL14*) was included in the qRT-PCR analysis for normalization of qRT-PCR expression levels. First-strand cDNA was synthesized using 1 μg of total RNA, oligo-dT, random hexamer primers and SuperScript III reverse transcriptase kit (Invitrogen Life Technologies, Carlsbad CA). Primers used for qPCR were designed using Primer Express (Applied Biosystems, Foster City, CA). The qRT-PCR assay was performed for each sample in duplicate wells using Power SYBR green PCR master mix (Applied Biosystems, Foster City, CA) and a gene-specific primer-pair (Sigma-Genosys, Woodlands, TX) on an ABI Prism Sequence Detection System 7900HT. Biogazelle qbase+ software [29] was used for pre-processing raw cycle threshold (Ct) data and the geNorm procedure for normalization of relative gene expression levels. The expression stability of candidate genes and a panel of invariant “housekeeping” genes was based on the geNorm (M) and coefficient of variation (CV) values across duplicate measurements of the 24 liver RNA samples. Ribosomal protein L14 (*RPL14*) and cytochrome c oxidase subunit VIIa polypeptide 2-like (*COX7A2L*) were selected as optimal co-reference genes with a mean of M = 0.251 and CV = 0.087. The PROC GLM in Statistical Analysis System (SAS, v.9.4; Cary NC) was used to analyze all pairwise differences among ontogenic stages and control for multiple comparisons using the option ADJUST = TUKEY. The Pearson’s correlation procedure in Excel was used to compare the average relative expression levels of each candidate DEG obtained from microarray analysis with that obtained by qRT-PCR analysis. Relative expression levels obtain by these two independent methods was verified by a significant (*P* ≤ 0.01) Pearson’s correlation coefficient (r).

## Results

### Initial visualization of gene expression patterns using GeneSpring® analysis

The preliminary GeneSpring analysis of the 24 Affymetrix arrays identified 4566 differentially-expressed genes (DEGs; FDR ≤ 0.01) in the contrast of 12 embryos vs.12 hatchling chicks. The unsupervised hierarchical clustering of these unfiltered DEGs by GeneSpring provided a visual image (heat map) of the most dramatic changes in hepatic gene expression during the peri-hatch period (Fig. 1a). This heat map shows three distinct patterns of hepatic gene expression during the embryo-to-hatchling transition. “Cluster A” DEGs are expressed at higher levels in embryos (E16, E18 and E20) and at lower levels after hatching, especially in fully-fed D3 and D9 hatchings. In contrast, the DEGs in “Cluster B” have a low abundance in embryos and sharply elevated expression in hatchlings, while “Cluster C” DEGs exhibit a mixed pattern of gene expression. The second panel (Fig. 1b), derived from k-means clustering, provides a higher resolution of gene expression patterns in Cluster A (1166 DEGs) and Cluster B (801 DEGs), with examples of two predominant genes per cluster. Deiodinase 3 (*DIO3*) and monoacylglycerol O-acyltransferase 1 (*MOGAT1*) are highly expressed in liver of late embryos, whereas thyroid hormone-responsive Spot 14 protein alpha (*THRSPA*) and deiodinase 2 (*DIO2*) were the most abundant DEGs found in liver of hatchling chicks. K-means clustering provides identification of individual genes and a high-resolution view of opposing expression patterns of the deiodinases (*DIO2, DIO3*), which clearly shows the pivotal role that thyroid hormone metabolism plays in the metabolic jump from ectotherm (embryos) to endotherm (hatchlings) during the peri-hatch period.

### Ingenuity pathway analysis (IPA): Identification of analysis ready DEGS

**Fig. 2:**
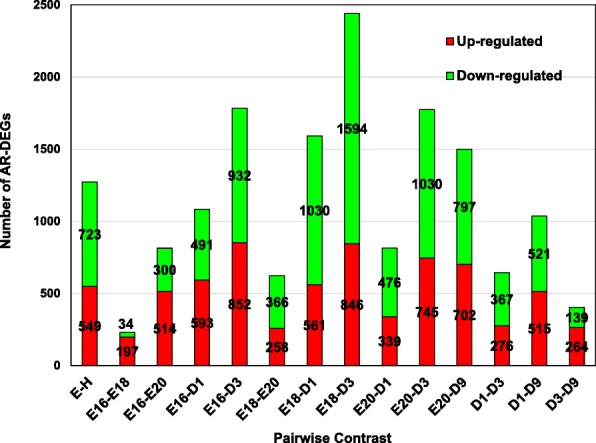
This stacked-bar graph provides the number of up-regulated (red portion of bar) and down-regulated (green portion of bar) AR-DEGs found by IPA in 14 pairwise contrasts of liver transcriptomes from embryos (E) and hatchlings (H). The list of annotated DEGs (FDR ≤ 0.05) for each developmental age was used to make 14 meaningful pairwise contrasts across embryos and hatchlings ages. An Excel file was generated for each contrast, and subsequently used as the input file for Ingenuity® Pathway Analysis (IPA). “Analysis Ready” (AR) genes are DEGs (AR-DEGs) that map to the Ingenuity® Knowledge Base. An annotated list of AR-DEGs for each of the 14 contrasts is provided in Additional file 3

The DEGs within each contrast were considered as “Analysis Ready” (AR-DEGs) if accrued and curated in the Ingenuity® Knowledge Base. The numbers of up-regulated and down-regulated AR-DEGs are presented for the14 pairwise contrasts in a split-bar graph (Fig. 2). An annotated list of AR-DEGs in each pairwise contrast is provided in Additional file 3. A primary contrast was made between all embryos (E) vs. all hatchlings (H), which identified 1272 AR-DEGs. The greatest number of AR-DEGs (2440 hepatic genes) was found in the E18 vs. D3 contrast, where 846 AR-DEGS were higher in E18 embryos (lipid-laden-ectotherms) and twice that number (1594 AR-DEGs) was found in liver of fully-fed D3 hatchlings. When E16 embryos were compared against either E18 (231AR-DEGs) or E20 (814 AR-DEGs) embryos, there were fewer genes compared to E16 embryos versus D1 (1084 AR-DEGs) or D3 (1784 AR-DEGs) hatchlings. The lowest number of AR-DEGs was found at ends of the ontogenic spectrum (Fig. 2), namely E16 vs. E18 (231 AR-DEGs) and D3 vs. D9 hatchlings (403 AR-DEGs).

**Fig. 3:**
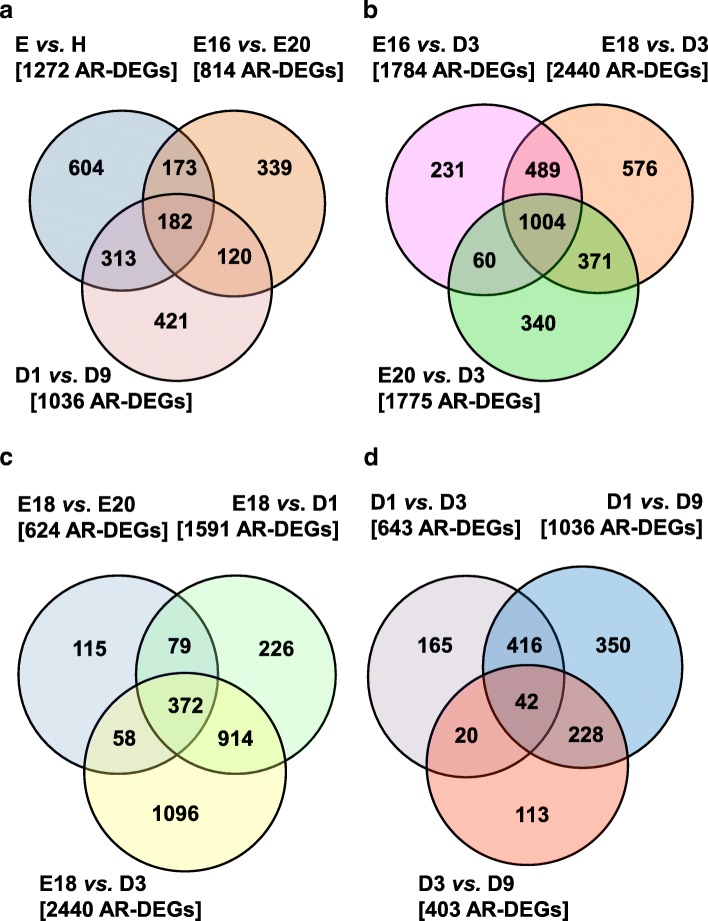
These Venn diagrams represent comparisons made from 12 meaning pairwise contrasts of hepatic transcriptomes across embryo and hatchling ages. The Venn diagrams show the number of unique genes and the number of commonly shared genes found in pairwise contrasts of hepatic transcriptomes. The Venn diagram in Panel **a** compares all embryos (E) vs. all hatchlings (H), E16 vs. E20 embryos, and D1 vs. D9 hatchlings. Panel **b** shows a Venn diagram comparing E16, E18 or E20 embryos vs. D3 hatchlings. Panel **c** shows the comparison of E18 embryos vs. E20, D1 or D3. Panel **d** illustrates shared and common genes among hatchling ages (D1 vs. D3, D1 vs. D9, and D3 vs. D9). The number of AR-DEGs found in each contrast is shown in brackets. The number within arcs represents genes commonly-shared between and among contrasts

Venn diagrams provide unique and commonly-shared sets of hepatic AR-DEGs among meaningful contrasts of embryo and hatchling ages (Fig. 3). The Venn diagram in Fig. 3a compared three contrasts: E vs. H, E16 vs. E20 embryos and D1 vs. D9 hatchlings. A group of 604 AR-DEGs were unique to the E vs. H contrast, which shared 173 AR-DEGs with the E16 vs. E20 contrast and 313 AR-DEGs with the D1 vs. D9 contrast. The Venn diagram in Fig. 3b shows larger numbers of AR-DEGs in contrasts made between each embryonic age (E16, E18 and E20) vs. fully-fed D3 hatchlings. Also, this Venn diagram contains the largest number of commonly-shared AR-DEGs (1004 genes) among contrasts between E16, E18 and E29 embryos compared with the D3 hatchlings. In Fig. 3c, E18 embryos were compared against E20 embryos, D1 or D3 hatchlings. Comparisons of E18 vs. E20, D1 or D3 (Fig. 3c) revealed 372 common genes and a large group of 1096 AR-DEGs that are unique to the E18 vs. D3 contrast and an additional 914 AR-DEGs that are shared with the E18 vs. D1 contrast. The fourth Venn diagram (Fig. 3d) represents exclusive contrasts of hatchlings. The D1 vs. D3 contrast (643 AR-DEGs) was made between newly-hatched, fasted chicks (D1) and fed D3 hatchlings. The D1 vs. D9 hatchling contrast yielded 1036 AR-DEGs, 416 genes were shared with the D1 vs. D3 contrast, and 228 AR-DEGs shared with the D3 vs. D9 contrast of 403 AR-DEGs. And as expected, this Venn diagram of hatchling contrasts had the lowest number (42 genes) of commonly-shared AR-DEGs.

### Ingenuity pathway analysis (IPA): Functional analysis of each pairwise contrast

#### Embryo (E) vs. hatchling (H) contrast

A description of the IPA of each pairwise contrast follows the same order in which the pair-wise contrasts are presented in Fig. 2 and Additional file 3. A brief summary of the IPA “Core Analysis” of 1272 AR-DEGs identified in the primary contrast of all embryos (E) vs. all hatchlings (H) is provided in Table 1. The top five canonical pathways over-represented by AR-DEGs from the E vs. H contrast were related to cholesterol biosynthesis, triacylglycerol biosynthesis, or activation of the ligand-activated nuclear receptor, retinoid X receptor (RXR) and its heterodimer partners [liver activated receptor (LXR) and farnesoid activated receptor (FXR)]. The top upstream regulator found in liver of embryos was peroxisome proliferator-activated receptor-alpha (*PPARA*), which is considered as the master regulator of lipid catabolism, namely lipolysis of yolk lipids. It should be noted that IPA estimates the top up-stream regulators according to the number of target AR-DEGs in the data set rather than whether or not the transcription factor (TF) itself was differentially expressed. PPARA was an AR-DEG that had 94 direct targets among the 1272 AR-DEGs. Sterol-response element binding factors 1 and 2 [SREBF1 (51 AR-DEG targets) and SREBF2 (28 AR-DEG targets) are ligand-activated transcription factors, which regulate sterol biosynthesis. Although not an AR-DEG itself, the tumor protein p53 (TP53) had the highest number of direct gene targets observed in the E vs. H contrast. Similarly, fork-head box O1 (*FOXO1*), a member of the fork-head family of transcription factors was not an AR-DEG in the E vs. H contrast, although IPA considered FOXO1 a top up-stream regulator due to its large number of direct target genes in this contrast. Actually, 23 transcription factors (TFs) were AR-DEGs in the E vs. H contrast, where 11 TFs were expressed higher in liver of embryos and 12 TFs were expressed higher in hatchlings (see Additional file 4). Of particular interest, a recently discovered gene SERTA domain containing 2 (*SERTAD2* or TRIP-Br2) was up-regulated in liver of embryos. *SERTAD2* is a transcription co-regulator of lipolysis, thermogenesis and oxidative metabolism in mammals.

**Table 1:** Ingenuity Pathway Analysis (IPA) of the liver transcriptome in the E vs. H contrast

Top Canonical Pathways	*p*-value	Overlap	Ratio
Super-pathway of Cholesterol Biosynthesis	1.00E-12	73.9%	17/23
Cholesterol Biosynthesis I-III	1.29E-08	90.0%	9/10
LXR/RXR Activation	1.24E-07	33.3%	24/72
FXR/RXR Activation	1.14E-07	31.6%	25/79
Triacylglycerol Biosynthesis	1.29E-06	43.8%	14/32
Top Upstream Regulators	*p*-value of overlap		# Target genes
PPARA	1.00E-26		94
SREBF1	5.15E-17		51
TP53	8.71E-16		186
SREBF2	2.88E-14		28
FOXO1	4.25E-12		74
Top Molecular and Cellular Functions	*p*-value		# Genes
Amino Acid Metabolism	1.97E-03 - 6.58E-14		72
Small Molecule Biochemistry	1.97E-03 - 6.58E-14		358
Lipid Metabolism	1.97E-03 - 2.36E-13		284
Molecular Transport	1.82E-03 - 2.36E-13		231
Vitamin and Mineral Metabolism	1.13E-03 - 3.43E-12		88
Physiological System Development and Function	*p*-value		# Genes
Tissue Morphology	1.50E-03 - 1.28E-06		186
Organismal Development	1.87E-03 - 1.63E-06		373
Organ Morphology	1.16E-03 - 4.30E-06		123
Renal and Urological System Development and Function	1.13E-03 - 4.30E-06		66
Embryonic Development	1.87E-03 - 1.19E-05		195
Toxicity Functions	*p*-value	# Genes	Ratio
LXR/RXR Activation	1.67E-07	32.9%	24/73
FXR/RXR Activation	2.14E-07	31.6%	25/79
Cholesterol Biosynthesis	2.69E-07	66.7%	10/15
LPS/IL-1 Mediated Inhibition of RXR Function	2.95E-05	22.1%	32/145
Liver Necrosis/Cell Death	5.92E-05	19.3%	42/218
Top Up-regulated genes	log2 Ratio (E/H)	Top Down-regulated genes	log2 Ratio (E/H)
MOGAT1	5.44	THRSPA	−7.15
DIO3	5.01	SLCO1A2	−5.82
PPM1K	4.65	ELOVL5	−5.60
PDK4	3.95	CDO1	−5.23
SLC13A3	3.82	FASN	−5.08
ITGBL1	3.66	SQLE	−4.92
TTLL2	3.64	DIO2	−4.84
ALKAL2	3.18	NSDHL	− 4.74
RET	3.16	CYP7A1	−4.73
ASCL1	3.11	AFMID	−4.44

Ingenuity Pathway Analysis (IPA) provided a functional analysis of 1272 “Analysis Ready” (AR) and differentially expressed genes (AR-DEGs) in the embryo vs. hatchling contrast. The *P*-values were derived by IPA from Fisher’s Exact Test, which indicates the likelihood of over-representation of AR-DEGs in particular canonical pathways and biological processes. The top 10 up-regulated genes in embryo (E) liver have positive E/H ratios, whereas the 10 down-regulated genes possess negative log2 ratios (E/H) which indicates higher expression in hatchling (H) liver

The top “Molecular and Cellular Functions”, found in the E vs. H contrast (Table 1), were “Small Molecule Biochemistry” (358 AR-DEGs), “Lipid Metabolism” (284 AR-DEGs), and “Molecular Transport” (231 AR-DEGs). Fifty AR-DEGs were functionally annotated by IPA as involved in “Amino Acid Metabolism”, where 28 genes were overexpressed in liver of embryos compared to 22 genes in this category expressed higher in hatchlings (Additional file 4). “Oxidation of Lipid” was another IPA “Biofunction” category that was over-represented by 40 metabolic AR-DEGs in the E (19 genes) vs. H (21 genes) contrast. “LPS/IL1 Mediated Inhibition of RXR Function” was another canonical pathway occupied by AR-DEGs from the embryo vs. hatchling contrast; 13 genes were more abundant in embryos and 19 genes were higher in liver of hatchlings. Hepatic genes involved in “Cholesterol Biosynthesis” are more numerous in liver of hatchlings (16/17 AR-DEGs), where only *HADHB* was expressed greater in embryos. “Triacylglycerol Synthesis” was also over- represented with 14 AR-DEGs, 6 genes (mainly acyltransferases) were higher in liver of embryos and 8 genes (3 acyltransferases and 3 fatty acid elongases) were expressed a greater levels in hatchlings. Four additional over-represent canonical pathways populated by AR-DEGs from the E vs. hatchling contrast included farnesoid X-activated receptor (“FXR)-RXR Activation” (25 AR-DEGs), liver activated receptor (“LXR-RXR Activation” (24 AR-DEGs), thyroid hormone receptor (“TR-RXR Activation” (16 genes), and the “Coagulation System” (5 genes expressed higher in embryos and one gene higher in hatchlings).

**Fig. 4:**
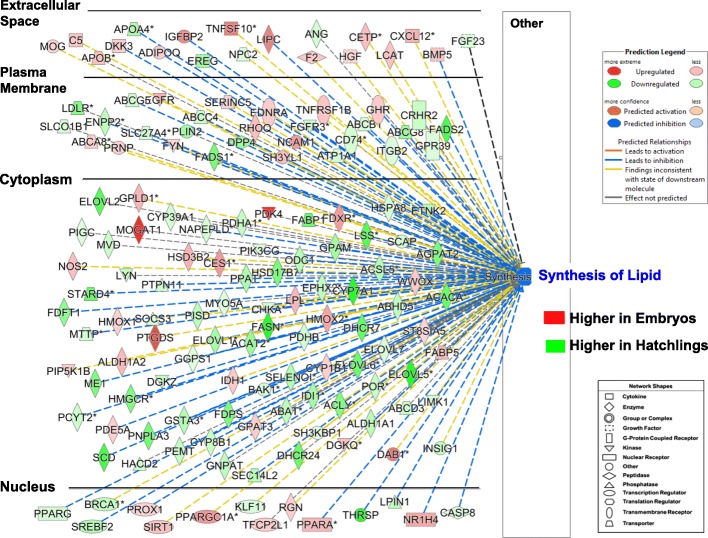
This figure provides the subcellular distribution of 149 hepatic AR-DEGs that regulate “Synthesis Lipid” from the contrast of all embryos vs. all hatchlings. Higher gene expression in embryos is indicated by red symbols and AR-DEGs with higher expression in liver of hatchlings have green symbols. However, the Ingenuity Upstream Regulator Analysis predicts that lipid synthesis would be inhibited in embryos as indicated by the blue label and blue dashed lines. This IPA prediction is based on actual E/H expression ratios and the observation that the number of AR-DEGs with higher expression in hatchlings (denominator) is 2-times greater than that of embryos (numerator). A small group of 14 transcription factors controls a much larger group of downstream metabolic genes. The largest number of AR-DEGs was found in “Cytoplasm”; these genes encode metabolic enzymes, kinases, phosphatases and transporters. The “Plasma Membrane” is mainly composed of genes encoding transmembrane and G-protein coupled receptors, transporters, enzymes, kinases and phosphatases. The extracellular AR-DEGs are a mixture of growth factors, adipokines, cytokines and transporters

The importance of hepatic lipid metabolism during the embryo-to-hatchling transition becomes clear by examining the subcellular distribution of 149 AR-DEGS that control “Lipid Synthesis” (Fig. 4.). This graphic overview shows higher expression of 54 AR-DEGs (red symbols) in the liver of embryos, while twice that number (95 AR-DEGs) were expressed higher in hatchlings (green symbols). In the nucleus, seven TFs were expressed higher in liver of embryos *PROX1*, *SIRT1*, *PPARGC1A*, *TFC2L1*, *RGN*, *PPARA* and *NR1H4*). Likewise, seven TFs were expressed higher in liver of hatchlings (*PPARG, SREBF2*, *BRCA1*, *KLF11*, *THRSPA*, *LPIN1* and *CASP8*). Thus, Ingenuity® Up-stream Regulator Analysis predicts that lipid synthesis was inhibited in liver of hatchlings when compared to the embryos (red symbols) as indicated by blue-dashed lines. However, the over-abundance of genes with green symbols (62 AR-DEGs) found in the cytoplasm are metabolic enzymes, transporters, kinases and phosphatases, which are actually expressed at higher levels in liver of hatchlings than in embryos (only 22 up-regulated genes). Among the most abundant cytosolic genes found in the embryo were several lipolytic AR-DEGs: *MOGAT1, PDK4*), *FDXR*, *LPL*, *HMOX2*, *PTGDS* and *DAB1*. Notable and highly-expressed lipogenic DEGs were found in the liver of hatchlings, including *SCD, FASN, ME1, AGPAT2, LSS, ACACA, ELOVL5* and *ELOVL6*. In the plasma membrane, 17 DEGs were expressed at high levels in hatchlings (i.e., *FADS1, FADS2, DDP4, PLIN2, ENPP2* and *LDLR*), while the *GHR*, *TNFRSF1B*, *ENDRA* and *EGFR* were upregulated in embryos. Extracellular space was occupied by 12 gene products expressed higher in the embryo (i.e., *APOB, ADIPOQ, IGFBP2, TNFSF10, IPC, F2, HGF, LCAT, CETP, CXCL12* and *BMP5*) when compared to only 5 hepatic gene products that were higher in extracellular space of the hatchlings (*APOA4, EREG, NPC2, ANG* and *FGF23*). This subcellular distribution of 149 AR-DEGs from the E vs. H contrast provides a comprehensive view of how 14 differentially-expressed TFs control lipid synthesis, while revealing major lipolytic genes in embryos and an even larger number of lipogenic genes in liver of hatchlings.

**Fig. 5:**
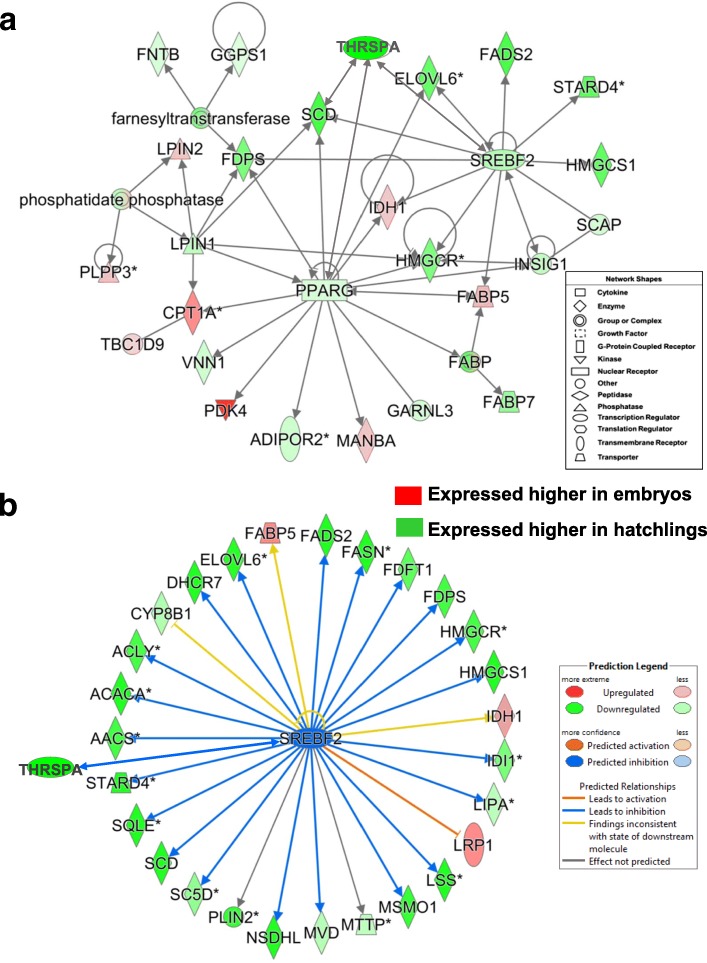
A gene network (Panel **a**) identified by IPA from the contrast of embryos (E) vs. hatchlings (H) that shows interaction of three lipogenic transcription factors (*THRSPA*, *PPARG, SREBF2*) with several direct target genes. This gene network was functionally annotated by IPA as involved in “Lipid Metabolism”. The legend provides a gene’s type while its relative expression (log2 ratio of E/H) indicates either higher expression in the embryo (red symbols) or a greater abundance in liver of hatchlings (green symbols). Panel **b**, Ingenuity® Upstream Regulator Analysis identified 28 direct targets of sterol response element binding factor 2 (*SREBF2*); most of which were expressed at higher levels in hatchlings. Ingenuity Upstream Regulator Analysis predicts inhibition of *SREBF2,* since 22 known direct targets of SREBF2 were downregulated (green symbols) in the E vs. H contrast. Actually, genes with green symbols are more abundant in liver of the hatchlings, since they have negative log2 E/H ratios

Another interaction network the E vs. H contrast was functionally annotated by IPA as “Lipid Metabolism, Molecular Transport” (Fig. 5). This network shows interactions among three lipogenic TFs (*THRSPA, PPARG* and *SREBF2*) and their direct target genes. The ligand-activated nuclear hormone receptor *PPARG* has a direct action on five target genes that were highly expressed in embryos (*IDH1*, *FABP5*, *MANBA*, *PDK4* and *CPT1A*). In addition, *PPARG* interacts with several genes that are more abundant in liver of hatchling chicks (*SCD, THRSPA, EVOLV6*, *HMGCR*, *FABP7*, *GARNl3*, *ADIPOR2*, *VNN1*, *LPIN1* and *FDPS*). LPIN1 interacts with LPIN2, *PLPP3* and *FDPS,* which has a direct action on *FNTB* and *GGPS1*. Six genes in this network have direct interactions with both *PPARG* and *SREBF*2 (*FDPS, SCD, THRSPA, ELOVL6, HMGCR* and *IDH1*). Numerous additional direct targets of *SREBF2* from the E vs. H contrast were identified by the Ingenuity® Upstream Regulator Analysis (Fig. 5b), which predicts that the majority of these 28 target genes would be inhibited (or down-regulated, as indicated by blue arrows) by *SREBF2*. Ingenuity only predicts that the LDL receptor related protein 1 (*LRP1*), which was up-regulated in embryos, would be inhibited by SREBP2 as indicated by the blunt orange line. However, two additional genes that were also highly abundant in the embryo, *IDH1* and *FABP5,* appeared to be inconsistent with IPA’s anticipated state. Interestingly, *SREBF2* and its direct target genes are involved in cholesterol metabolism and lipogenesis in particular, which are both enhanced in liver of hatchling chicks. As direct targets of SREBF2, only *LRP1, IDH1* and *FABP5* were up-regulated in liver of embryos. Furthermore, 26 of the most abundant AR-DEGs found in liver of hatchling chicks (Additional file 4) are involved in either cholesterol biosynthesis (i.e., *MSMO1*, *MVD*, *CYP8B1* and *DHCR7*) or lipogenesis (i.e., *FADS2, FASN, HMGCS1, LPIN2* and *ACACA*)*.*

**Fig. 6:**
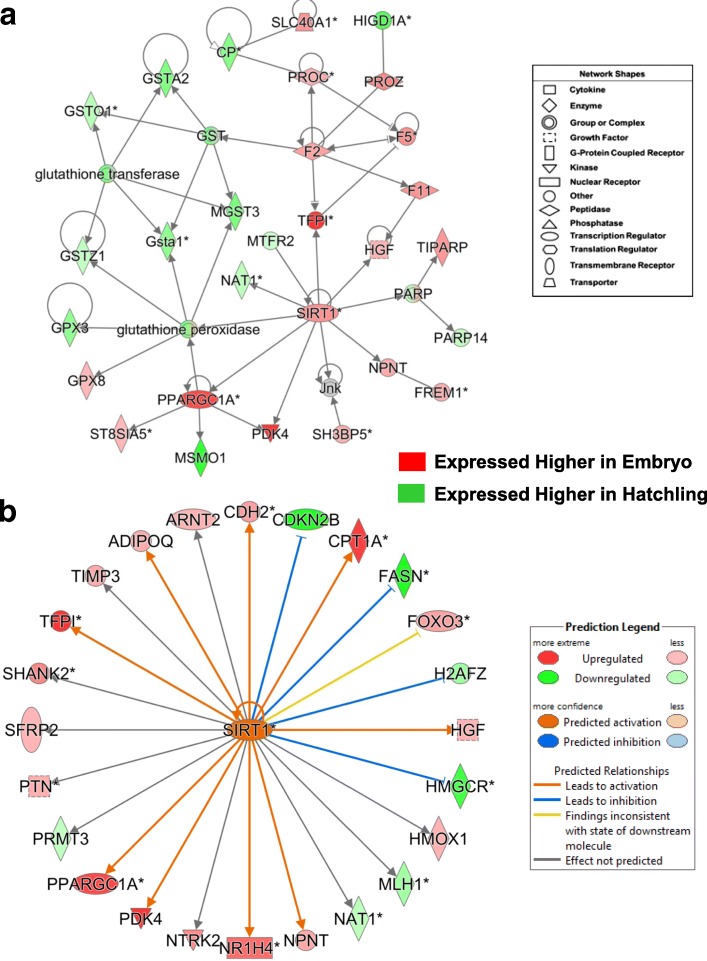
A gene interaction network identified by IPA from the E vs. H contrast and functionally annotated as “Hematological System Development and Function”. This gene network (Panel **a**) is centered on direct interactions of two transcription regulators [PPARG coactivator 1 alpha (*PPARGC1A*) and sirtuin 1 (*SIRT1*)] with several blood clotting factors [tissue factor pathway inhibitor (*TFPI*); coagulation factor II, thrombin (*F2*); coagulation factor XI (*F11*); protein C, in-activator of coagulation factors Va and VIIIa (*PROC*); and protein Z, vitamin K-dependent plasma glycoprotein (*PROZ*)] and with components of the glutathione transferase pathway (*GSTA1, GSTA2, GSTO1, GSTZ1, MGST3, GPX3* and *GPX8*). Panel **b** shows 24 direct targets of SIRT1 that were identified by Ingenuity® Up-stream Regulator Analysis, which predicts activation of *SIRT1* since 17 of its known direct targets are upregulated in embryos. Orange arrows show genes that are up-regulated by *SIRT1*, while blunt blue edges indicate inhibition of known target genes, as predicted by Ingenuity software. Blue blunt lines predicts that activated *SIRT1* would lead to inhibition of four known targets [cadherin 2 (*CDH2*) or inhibition [cyclin dependent kinase inhibitor 2B (*CDKN2B*); fatty acid synthase (*FASN*)*;* H2A histone family member Z (*H2AFZ*); and 3-hydroxy-3-methylglutaryl-CoA reductase (*HMGCR*)]. Dark grey arrows identify several *SIRT1* target genes, where an effect could not be predicted by IPA

The gene interaction network in Fig. 6a, found in the E vs. H contrast, was functionally annotated by IPA as “Hematological System Development and Function”. This network was centered on interaction of several blood clotting factors (*TFPI*, *F2*, *F11*, *PROC* and *PROZ*) with two lipolytic TFs (*PPARGC1A* and *SIRT1*) and with components of the glutathione transferase pathway (*GSTA1, GSTA2, GSTO1, GSTZ1, MGST3, GPX3* and *GPX8*). Ingenuity® Up-stream Regulator Analysis also identified 24 target genes of SIRT1 (Fig. 6b), which would either activate several genes in embryos (*CPT1A, HGF*, *NR1H4*, *PDK4, PPARGC1A, TFP1, ADIPOQ* and *CDH2*) or inhibit other genes in (*CDKN2B*, *FASN, H2AFZ* and *HMGCR*). These genes are highly expressed in liver of embryos and directed at lipolysis and gluconeogenesis, while the AR-DEGs with green symbols are mainly lipogenic genes.

#### E16 vs. E18 contrast

The E16 vs E18 contrast yielded the smallest number (231) of AR-DEGs (Fig. 2; Additional file 3), which prevented generation of highly-populated gene interaction networks by IPA. Interestingly, the most abundant DEGs expressed in the liver of E16 embryos when compared to E18 embryos were seven transcripts that correspond to feather keratin (*FKER*). Unfortunately, these avian-specific transcripts were rejected by Ingenuity software and not included in the IPA functional analysis of the E16 vs. E18 contrast.

#### E16 vs. E20 contrast

The contrast of E16 vs. E20 embryos identified 814 AR-DEGs, which included 514 genes up-regulated in E16 liver (Additional file 3). A summary of the IPA of the liver transcriptome of E16 vs. E20 embryos is presented in Table 2. The top five canonical pathways over-populated by 814 AR-DEGs in this contrast were “LPS/IL-1 Mediated Inhibition of RXR Function, Mitotic Roles of Polo-Like Kinase, Complement System, Tyrosine Degradation I, and Cell Cycle Control of Chromosomal Replication”. The top upstream regulators identified by IPA from this contrast were *E2F4*, *TCF3*, *PPARA*, *E2F1* and *TCF4*. The top “Molecular and Cellular Functions” recognized in the E16 vs. E20 contrast were “Cell Cycle”, “Cellular Assembly and Organization”, “DNA Replication, Recombination, and Repair”, “Cell Death and Survival”, and “Cell Morphology”. The top “Physiological Function” category included the “Reproductive” and “Digestive” systems, “Organ Morphology”, “Organismal Development” and “Behavior”. Under “Toxicity Functions”, IPA assigned several AR-DEGs to “Inhibition of RXR Function”, fatty acid metabolism, renal failure, and transmembrane potentials. Interestingly, the hepatic deiodinases were the top 10 opposing DEGs discovered in the liver of E16 (*DIO3*; + 3.6 log2 ratio) vs. E20 (*DIO2*; − 7.2 log2 ratio) embryos. Another gene highly expressed in liver of E16 embryos was angiopoietin-like protein 3 (*ANGPTL3*), a regulator of lipoprotein lipase. Serpin family A member 3 (*SERPINA3*), *HPX*, *FABP1* and *KLF9* were DEGs that were up-regulated in liver of E20 embryos. Of particular interest, the E18 vs. E20 contrast revealed higher expression of three Kruppel-like factors (*KLF9*, log2–3.52; *KLF11*, log2–0.85; *KLF13*, log2–2.31) in liver of E20 embryos (Additional file 3).

**Table 2:** IPA summary of the liver transcriptome in the E16 vs. E20 contrast

Top Canonical Pathways	*p*-value	Overlap	Ratio
LPS/IL-1 Mediated Inhibition of RXR Function	4.21E-06	17.9%	24/134
Mitotic Roles of Polo-Like Kinase	8.94E-06	26.5%	13/49
Complement System	9.91E-06	37.5%	9/24
Tyrosine Degradation I	8.12E-05	80.0%	4/5
Cell Cycle Control of Chromosomal Replication	1.23E-04	23.9%	11/46
Top Upstream Regulators	*p*-value of overlap		# Target genes
E2F4	2.52E-23		52
TCF3	1.14E-19		56
PPARA	1.54E-15		57
E2F1	1.98E-15		62
TCF4	6.52E-14		36
Top Molecular and Cellular Functions	*p*-value		# Genes
Cell Cycle	1.35E-03 - 7.51E-18		212
Cellular Assembly and Organization	1.02E-03 - 7.51E-18		118
DNA Replication, Recombination, and Repair	1.02E-03 - 7.51E-18		150
Cell Death and Survival	1.33E-03 - 1.54E-11		350
Cell Morphology	2.22E-04 - 1.71E-11		74
Physiological System Development and Function	*p*-value		# Genes
Reproductive System Development and Function	5.11E-04 - 2.03E-06		23
Digestive System Development and Function	4.37E-04 - 7.57E-05		18
Organ Morphology	4.37E-04 - 7.57E-05		21
Organismal Development	4.37E-04 - 7.57E-05		142
Behavior	1.52E-04 - 1.52E-04		18
Top Toxicity Functions	*p*-value	# Genes	Ratio
LPS/IL-1 Mediated Inhibition of RXR Function	1.70E-05	16.6%	24/145
Fatty Acid Metabolism	6.31E-05	21.2%	14/66
Cell Cycle: G2/M DNA Damage Checkpoint Regulation	2.09E-04	24.4%	10/41
Genes Upregulated in Response to Chronic Renal Failure	1.02E-03	75.0%	3/4
Transmembrane Potential of Mitochondria and Membrane	1.50E-03	16.5%	13/79
Top Up-regulated Genes	log2 Ratio	Top Down-regulated Genes	log2 Ratio
DIO3	3.65	DIO2	−7.19
ACMSD	3.65	SERPINA3	−5.55
ANGPTL3	3.62	HPX	−4.61
HDAC9	3.35	FKBP5	−4.37
GAS2	3.19	CA4	−4.20
CHODL	3.11	FABP1	−4.07
CKS2	3.02	KLF9	−3.64
CKAP2	2.97	IP6K2	− 3.61
AURKA	2.93	SLCO1A2	−3.60
KPNA2	2.90	ITIH3	−3.49

Ingenuity Pathway Analysis (IPA) analysis of 814 “Analysis Ready” (AR) and differentially expressed (DE) genes (AR-DEGs) from the E16 vs. E20 contrast. The top 10 up-regulated genes in E16 liver have positive log2 ratios (E16/E20), whereas the 10 down-regulated genes possess negative log2 ratios (E16/E20), which indicates higher expression in liver of E20 embryos

#### E16 vs. D1 contrast

The contrast of E16 embryos vs. D1 hatchlings recognized 1084 AR-DEGs, where 593 AR-DEGs were more abundant in liver of the E16 embryos (Fig. 2; Additional file 3). The highest DEGs found in embryo liver from the E16 vs. D1 contrast included *DIO3* and *MOGAT1*, while *DIO2* and *FABP1* were among the highest AR-DEGs found in fasted D1 hatchlings.

#### E16 vs. D3 contrast

The E16 vs. D3 contrast identified 1784 AR-DEGs with 852 transcripts up-regulated in liver of E16 embryos compared with 932 AR-DEGs in D3 hatchlings. *DIO3* and *MOGAT1* were also among the highest DEGs in E16 embryos from the E16 vs. D3 contrast whereas *DIO2* and *THRSPA* were overexpressed DEGs found in liver of D3 hatchlings.

#### E18 vs. E20 contrast

A total of 624 AR-DEGs were identified by the E18 vs. E20 contrast, where 258 AR-DEGs were expressed at higher levels in E18 embryos compared to 366 AR-DEGs overexpressed in the E20 embryos (Table 3). The major canonical pathways highly populated by AR-DEGs from this contrast were “LPS/IL-1 Mediated Inhibition of RXR Function, Mitotic Roles of Polo-Like Kinase, Protein Ubiquitination Pathway, Unfolded Protein Response”, and “Tyrosine Degradation I”. The top five upstream regulators found in the E18 vs. E20 contrast included *PPARA*, *TCF3*, *ATF6*, *FOXO3* and *RARA*. “Small Molecule Biochemistry” and “Cell Cycle” were the highly represented molecular and cellular functions according to IPA. Likewise, “Organ Morphology” and “Organismal Development” were highly populated physiological functions identified by IPA. Among the top toxicology categories were “Fatty Acid Metabolism”, inhibition/activation of RXR and “Cell Cycle”. Similar to the E16 vs. E18 contrast, the thyroid hormone deiodinases were the most abundant DEGs in E18 (*DIO3*; log2 + 3.8) vs. E20 (*DIO2*; log2–5.6) contrast. Several additional genes, highly expressed in the E20 liver, are involved in lipid metabolism (*SERPINA3, HMGCS1* and *KLF9*) or fatty acid transport (*FABP1*). Lists of functionally-annotated hepatic AR-DEGs belonging to these canonical pathways and biological functions are provided in worksheets of Additional file 5. “Lipid Synthesis” was a subcategory under “Lipid Metabolism” that was over-populated by 60 AR-DEGs; 18 DEGs were up-regulated in E18 embryos, whereas 42 hepatic genes were expressed at higher levels in liver of E20 embryos. Under “Amino Acid Metabolism”, 21 AR-DEGs were more abundant in E18 embryos, while 12 genes were expressed higher in E20 embryos. Under the “Insulin Resistance” category, IPA identified 12 AR-DEGs that were more abundant in E18 embryo liver, while 16 genes were more abundant in E20 than in E18 embryos. Nineteen AR-DEGs were assigned by IPA to the “Sirtuin Signaling” pathway, where 14 AR-DEGs were higher in E20 embryos. The “FXR-RXR Activation” pathway was occupied by 11 AR-DEGs, where 7 genes were more abundant in E20 embryos.

**Table 3:** IPA summary of the liver transcriptome in the E18 vs. E20 contrast

Top Canonical Pathways	*p*-value	Overlap	Ratio
LPS/IL-1 Mediated Inhibition of RXR Function	1.11E-04	13.4%	18/134
Mitotic Roles of Polo-Like Kinase	1.22E-04	20.4%	10/49
Protein Ubiquitination Pathway	4.53E-04	10.9%	22/202
Unfolded protein response	9.41E-04	19.0%	8/42
Tyrosine Degradation I	1.14E-03	60.0%	3/5
Top Upstream Regulators	*p*-value of overlap		# Target genes
PPARA	6.04E-14		47
TCF3	1.11E-11		38
ATF6	1.56E-08		13
FOXO3	5.68E-08		32
RARA	6.84E-08		30
Top Molecular and Cellular Functions	*p*-value		# Genes
Amino Acid Metabolism	2.43E-03 - 1.01E-12		42
Small Molecule Biochemistry	3.58E-03 - 1.01E-12		161
Cell Cycle	3.01E-03 - 5.43E-10		82
Cell Morphology	2.92E-03 - 1.29E-09		39
Cellular Assembly and Organization	2.92E-03 - 1.29E-09		43
Physiological System Development and Function	*p*-value		# Genes
Hematological System Development and Function	2.85E-03 - 2.16E-05		23
Lymphoid Tissue Structure and Development	2.43E-03 - 2.16E-05		17
Organ Morphology	2.43E-03 - 2.16E-05		55
Tissue Morphology	2.43E-03 - 2.16E-05		25
Organismal Development	3.07E-03 - 2.64E-05		68
Top Toxicity Functions	*p*-value	Overlap	Ratio
Fatty Acid Metabolism	7.84E-05	18.2%	12/66
LPS/IL-1 Mediated Inhibition of RXR Function	2.72E-04	12.4%	18/145
FXR/RXR Activation	1.62E-03	13.9%	11/79
Cell Cycle: G2/M DNA Damage Checkpoint Regulation	3.55E-03	17.1%	7/41
PXR/RXR Activation	3.55E-03	17.1%	7/41
Top Up-regulated Genes	log2 Ratio	Top Down-regulated Genes	log2 Ratio
DIO3	3.81	DIO2	−5.61
ANGPTL3	3.14	SERPINA3	−4.54
SLC2A5	2.99	FABP1	−3.93
RET	2.83	HIGD1A	−3.81
GAS2	2.70	HMGCS1	−3.78
HDAC9	2.69	KLF9	−3.52
NAV2	2.47	C6	−3.44
CHODL	2.46	HPX	−3.38
SLC13A3	2.45	FKBP5	−3.28
SOX9	2.36	SLCO1A2	−3.26

Ingenuity Pathway Analysis (IPA) was used for functional analysis of 624 AR-DEGs identified in the E18 vs. E20 contrast (E18/E20 ratio). AR-DEGs with positive log2 ratios are expressed higher in liver of E18 embryos, while negative log 2 ratios indicate higher expression in E20 embryos

**Fig. 7:**
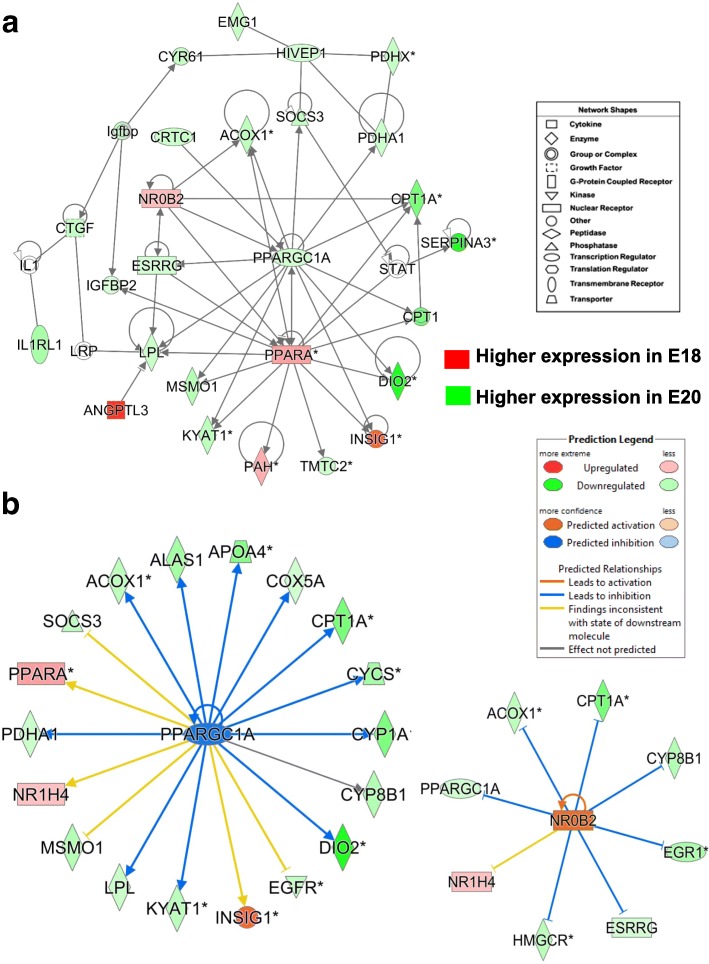
This gene network (Panel **a**) from the E18 vs. E20 contrast was functionally annotated by IPA as “Lipid Metabolism”. It shows interactions among four nuclear hormone receptors [nuclear receptor subfamily 0 group B member 2 (*NROB2*), estrogen related receptor gamma (*ESSRG*), peroxisome proliferator-activated receptor-alpha (*PPARA*) and *PPARGC1A*] that regulate energy balance and lipid metabolism in liver of late embryos. In Panel **b**, Ingenuity® Upstream regulator Analysis predicts that *PPARGC1A* itself is inhibited (blue symbol) because 16 known target genes are down-regulated in the E18/E20 contrast. The blue arrows predict that 11 known target genes should be inhibited. Actually, red-colored gene symbols indicate higher expression in liver of E18 embryos, whereas the green-colored symbols show higher expression in E20 embryos. In addition, Ingenuity predicts that activated *NROB2* leads to inhibition (blunt blue lines) of seven lipogenic genes (*PPARGC1A, ANOX1, CPT1A, CTP8B1, EGR1, ESRRG* and *HMGCR*), whereas, the upregulation of *NR1H4* is inconsistent with its expected state (based on the Ingenuity Knowledge Base)

This gene interaction network (Fig. 7) from the E18 vs. E20 contrast was functionally annotated by IPA as “Lipid Metabolism”. In liver of late chicken embryos, the interactions among four nuclear hormone receptors (*NROB2*, *ESRRG*, *PPARA* and *PPARGC1A*) control energy balance and lipid metabolism. Ingenuity predicts PPARGC1A itself is inhibited (blue symbol) and blue arrows show inhibition of target genes in the E18 vs. E20 embryos (Fig. 7b). Fifteen direct targets of *PPARGC1A* (green symbols) are highly expressed in liver of E20 embryos, whereas red-colored gene symbols indicate up-regulated expression in liver of E18 embryos. These genes (i.e., *INSIG1, NR1H4* and *PPARA*) are involved in fat catabolism, while genes with green-colored symbols indicate higher expression of lipogenic genes in E20 embryos. In addition, Ingenuity predicts activation of the orphan receptor *NROB2* (nuclear receptor subfamily 0 group B member 2), which leads to inhibition (blunt blue lines) of seven lipogenic genes (*PPARGC1A, ANOX1, CPT1A, CTP8B1, EGR1, ESRRG* and *HMGCR*). Another direct target of *NROB2* (an inhibitor of estrogen, thyroid and retinoid nuclear hormone receptors) was the ligand-activated transcription factor *NR1H4*, which is activated by bile acids and thereby controls expression of genes involved in bile acid synthesis and transport. NR1H4 is also a direct target of PPARGC1A.

#### E18 vs. D1 contrast

The functional analysis of 1591 AR-DEGs found in the E18 vs. D1 contrast is presented in Table 4. The major canonical pathways identified by IPA in this contrast were “NRF2-mediated Oxidative Stress Response” and “Hereditary Breast Cancer Signaling”. The largest number of direct target genes was associated with two TFs, TP53 and ESR1. The top “Molecular and Cellular Functions” recognized by IPA of the E18 vs. D1 contrast were “Cell Cycle”, “Cellular Assembly and Organization”, “DNA Replication, Recombination, and Repair”, “Cell Death and Survival”, and “Lipid Metabolism”. Under the category “Physiological System Development and Function”, the largest number of DEGs was assigned to “Embryonic Development”. The top “Toxicity Functions” were related to the oxidative stress response, liver necrosis/proliferation and RXR activation. The highest DEGs in liver of E18 embryos were *DIO3* and *MOGAT1*, while in D1 hatchling chicks the most abundant transcripts were involved in lipid metabolism (*DIO2, ELOVL5, FABP1* and *HMGCS1*). Furthermore, four members of the Kruppel-like transcription factors (*KLF9, KLF11, KLF13* and *KLF15*) were abundant DEGs found in liver of fasted D1 hatchlings (Additional file 3).

**Table 4:** IPA summary of the liver transcriptome in the E18 vs. D1 contrast

Top Canonical Pathways	*p*-value	Overlap	Ratio
Cell Cycle Control of Chromosomal Replication	9.58E-07	41.3%	19/46
Mitotic Roles of Polo-Like Kinase	5.36E-05	34.7%	17/49
NRF2-mediated Oxidative Stress Response	5.68E-05	24.3%	36/148
Role of BRCA1 in DNA Damage Response	6.89E-05	30.4%	21/69
Hereditary Breast Cancer Signaling	2.05E-04	24.8%	29/117
Top Upstream Regulators	*p*-value of overlap		# Target genes
TP53	3.51E-20		228
E2F4	1.61E-17		63
E2F1	5.07E-17		96
ESR1	2.04E-14		193
TCF3	1.50E-12		67
Top Molecular and Cellular Functions	*p*-value		# Genes
Cell Cycle	1.11E-03 - 8.65E-14		366
Cellular Assembly and Organization	1.11E-03 - 8.65E-14		276
DNA Replication, Recombination, and Repair	1.11E-03 - 8.65E-14		265
Cell Death and Survival	1.07E-03 - 4.85E-13		626
Lipid Metabolism	1.11E-03 - 1.49E-07		252
Physiological System Development and Function	*p*-value		# Genes
Embryonic Development	1.11E-03 - 1.20E-05		140
Organismal Survival	1.20E-05 - 1.20E-05		36
Reproductive System Development and Function	5.21E-04 - 1.87E-05		27
Hematological System Development and Function	1.09E-03 - 5.24E-05		27
Hematopoiesis	1.09E-03 - 5.24E-05		21
Top Toxicity Functions	*p*-value	Overlap	Ratio
NRF2-mediated Oxidative Stress Response	7.48E-06	25.2%	41/163
Liver Necrosis/Cell Death	2.94E-04	20.9%	46/220
Liver Proliferation	7.86E-04	21.1%	38/180
FXR/RXR Activation	1.43E-03	24.3%	20/79
LXR/RXR Activation	3.31E-03	24.7%	18/73
Top Up-regulated Genes	log2 Ratio	Top Down-regulated Genes	log2 Ratio
DIO3	4.82	SLCO1A2	−6.69
SLC13A3	4.67	DIO2	−5.44
MOGAT1	4.44	ELOVL5	−4.96
RET	4.37	COCH	−4.96
TTLL2	4.25	FABP1	−4.63
ASCL1	3.94	HIGD1A	−4.63
PPM1K	3.93	HPS5	−4.63
ALKAL2	3.76	SLC51A	−4.60
TFPI	3.47	CYP7A1	−4.54
ATP2B2	3.35	HMGCS1	−4.46

Ingenuity Pathway Analysis (IPA) was used for functional analysis of 1591 AR-DEGs identified in the E18 vs. D1 contrast (E18/D1). AR-DEGs with positive log2 ratios are expressed higher in liver of E18 embryos, whereas negative log2 ratios indicate higher expression in D1 hatchlings

#### E18 vs. D3 contrast

The largest number of AR-DEGs was found in the E18 vs. D3 contrast (2440 AR-DEGs), which overpopulated the cholesterol and triacylglycerol biosynthetic pathways, and the “Intrinsic Prothrombin Activation Pathway” where all 12 AR-DEGs were up-regulated in liver of E18 embryos (Table 5). The top “Up-stream Regulators” in the E18 vs. D3 contrast included TP53, PPARA, and FOXO3. Among the top “Molecular and Cellular Functions” identified by IPA were the “Cell Cycle”, “DNA Replication, Recombination, and Repair”, “Small Molecule Biochemistry” (535 AR-DEGs; including 215 AR-DEGs that belong to the “Lipid Synthesis” subcategory; see Additional file 6), and “Cellular Assembly and Organization”. “Organismal Survival” was the most populated physiological function found with this contrast. The top “Toxicity Functions” assigned to AR-DEGs from the E18 vs. D3 include “Cholesterol Biosynthesis”, NRF2-Mediated Oxidative Stress Response, “LXR/RXR Activation”, and “TR/RXR Activation”. The top 10 “Up-regulated Genes” in E18 embryos were *DIO3, MOGAT1* and *PDK4*, whereas lipogenic genes (*THRSPA, SQLE, ELOVL5, DIO2*, *FASN* and *SCD*) were highly expressed in liver of fully-fed D3 hatchlings. The E18 vs. D3 contrast revealed seven members of the Kruppel-like transcription factor family as AR-DEGs (Additional files 3 and 6).

**Table 5:** IPA summary of the liver transcriptome in the E18 vs. D3 contrast

Top Canonical Pathways	*p*-value	Overlap	Ratio
Superpathway of Cholesterol Biosynthesis	2.07E-08	73.9%	17/23
Cholesterol Biosynthesis I-III	3.03E-06	90.0%	9/10
Cell Cycle Control of Chromosomal Replication	4.22E-05	45.7%	21/46
Intrinsic Prothrombin Activation Pathway	6.93E-05	60%	12/20
Triacylglycerol Biosynthesis	8.97E-05	50%	16/32
Top Upstream Regulators	*p*-value of overlap		# Target genes
TP53	1.00E-21		318
PPARA	5.67E-18		115
E2F1	9.13E-17		125
E2F4	5.54E-15		75
FOXO3	3.82E-13		87
Top Molecular and Cellular Functions	*p*-value		# Genes
Cell Cycle	1.57E-03 - 2.09E-10		499
DNA Replication, Recombination, and Repair	1.38E-03 - 2.09E-10		345
Amino Acid Metabolism	1.57E-03 - 2.48E-10		79
Small Molecule Biochemistry	1.57E-03 - 2.48E-10		535
Cellular Assembly and Organization	1.38E-03 - 6.63E-10		326
Physiological System Development and Function	*p*-value		# Genes
Organismal Survival	9.34E-04 - 8.88E-06		682
Embryonic Development	1.23E-03 - 3.36E-05		90
Tissue Morphology	1.57E-03 - 3.36E-05		207
Hematological System Development and Function	1.38E-03 - 1.83E-04		114
Organismal Development	1.30E-03 - 1.83E-04		191
Top Toxicity Functions	*p*-value	Overlap	Ratio
Cholesterol Biosynthesis	8.13E-05	66.7%	10/15
NRF2-mediated Oxidative Stress Response	1.60E-04	31.3%	51/163
Liver Necrosis/Cell Death	2.67E-04	29.1%	64/220
LXR/RXR Activation	3.06E-04	37.0%	27/73
TR/RXR Activation	1.01E-03	34.6%	27/78
Top Up-regulated Genes	log2 Ratio	Top Down-regulated Genes	log2 Ratio
DIO3	6.62	THRSPA	−8.86
MOGAT1	6.38	SLCO1A2	−6.78
PDK4	5.35	SQLE	−6.71
PPM1K	5.32	CDO1	−6.64
RET	4.59	ELOVL5	−6.64
ITGBL1	4.43	DIO2	−6.64
ALKAL2	4.37	FASN	−6.48
TTLL2	4.14	NSDHL	−6.24
SLC13A3	4.05	SCD	−5.71
DNAJC12	3.89	CYP7A1	−5.65

Ingenuity Pathway Analysis (IPA) was used for functional analysis of 2440 AR-DEGs identified in the E18 vs. D3 contrast (E18/D3 ratio). AR-DEGs with positive log2 ratios are expressed higher in liver of E18 embryos, while negative log 2 ratios indicate higher expression in D3 hatchlings

**Fig. 8:**
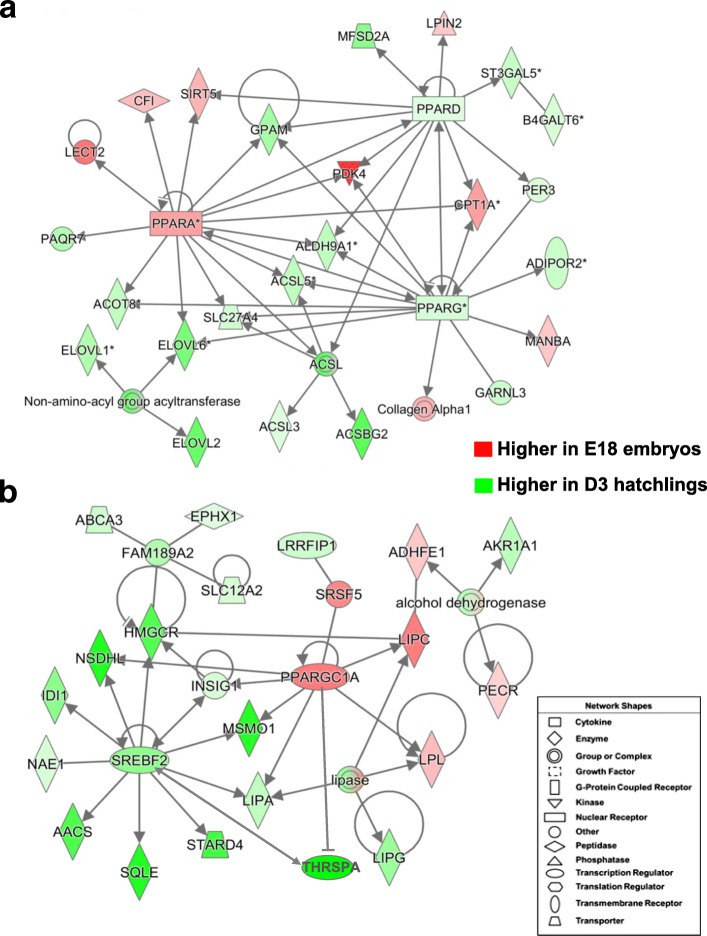
These two gene interaction networks were derived from a subset of 1004 commonly-shared genes found by comparing three contrasts (E16, E18 or E20 embryos vs. D3 hatchlings (see Venn diagram in Fig. 3b). Both networks were overlaid with expression values from the E18 vs. D3 contrast as indicated by the legend. This subset of commonly-shared AR-DEGs was enriched with multiple TFs and their direct target genes and functionally annotated by IPA as ‘Lipid Metabolism, Molecular Transport, Small Molecule Biochemistry’. The top panel (**a**) shows the interaction of three major TFs (*PPARA, PPARG* and *PPARD*) with several common target genes. The second gene network (Panel **b**) shows the interaction of three TFs (*SREBF2, PPARGC1A* and *THRSPA*). *SREBF2* and its direct target genes, including *THRSPA*, were expressed higher in liver of D3 hatchlings. *PPARGC1A* and *THRSPA* appear to have opposing actions on lipid metabolism as indicated by the blunt edge

Two gene interaction networks depicted in Fig. 8 were derived from IPA of a subset of 1004 commonly-shared genes identified by the Venn diagram shown in Fig. 3b, which compared contrasts of E16, E18 or E20 embryos vs. D3 hatchlings. This subset of commonly-shared AR-DEGs, enriched with multiple TFs and their direct target genes, was functionally annotated by IPA as ‘Lipid Metabolism, Molecular Transport, Small Molecule Biochemistry’ (see Additional file 3; last worksheet). The top panel (Fig. 8a) shows the interaction of three TFs (*PPARA, PPARG* and *PPARD*) with several common target genes (*SIRT5, GPAM, PDK4, ALDH9A, ACSL, SLC27A4, ELOVL6, ACOT8, CPT1A* and *PER3*). The ligand-activated TF *PPARA* and its five direct targets (*CPT1A, SIRT5, CFI* and *LECT*) were expressed at higher levels in liver of E18 embryos. In contrast, *PPARG* and *PPARD* and 11 target genes are expressed higher in liver of D3 hatchlings, to support lipogenesis and energy production of these fully-fed hatchlings. *PPARG* has four unique direct target genes (*ADIPOR2*, *MANBA, GARNL3* and the collagen alpha 1 complex), whereas *PPARD* has only four unique targets (*MFSD2A, LPIN2* and *ST3GAL5*) in this gene network. The second gene network (Fig. 8b) shows the interaction of two major lipogenic TFs (*THRSPA* and *SREBF2*; both expressed higher in D3 hatchlings) with the transcriptional coactivator *PPARGC1A,* which was expressed higher in liver of E18 embryos. Only five genes, directly or indirectly responsive to PPARGC1A were expressed at higher in liver of E18 embryos, whereas *SREBF2* and its 11 target genes, including *THRSPA*, were more abundant in liver of D3 hatchlings. Most of the target genes of *SREBF2* are involved in metabolism of cholesterol, sterol and ketones.

**Fig. 9:**
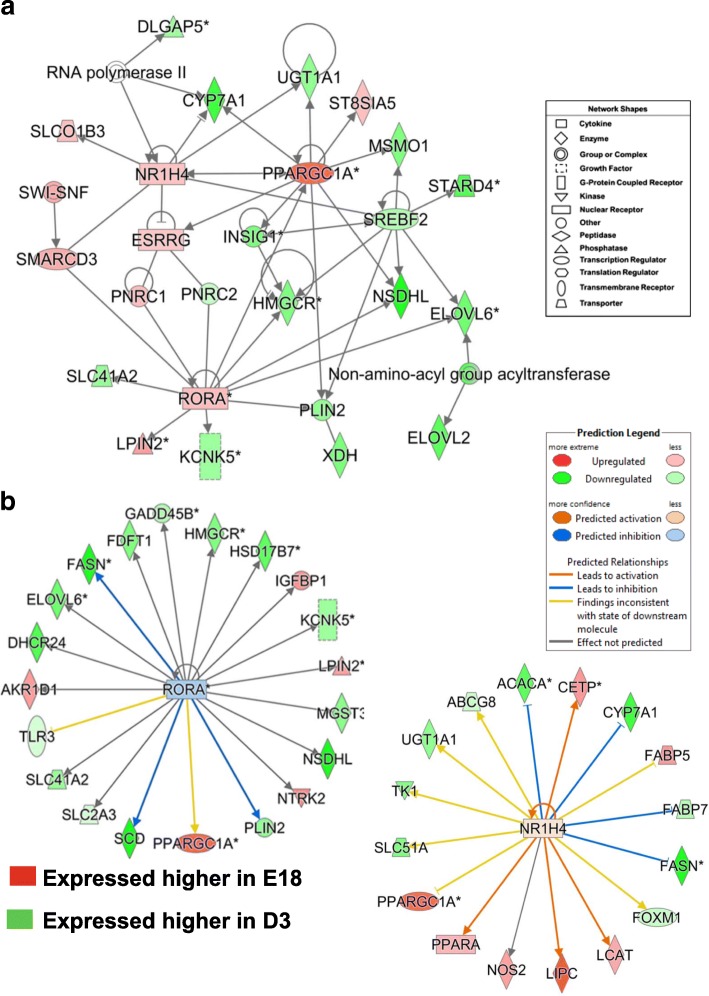
Another gene network, functionally annotated by IPA as “Lipid Metabolism”, was identified in E18 vs. D3 contrast (**a**). This network shows interactions among six up-stream regulators [RAR related orphan receptor A (*RORA*), SWI/SNF related, matrix associated, actin dependent regulator of chromatin, subfamily d, member 3 (*SMARCD3*), *ESRRG*, *NR1H4* (or farnesoid X receptor, *FXR*), *PPARGC1A* and *SREBF2*]. Ingenuity Upstream Regulator Analysis predicts inhibition of *RORA* (Panel **b**), since the majority of its target genes are down-regulated (green symbols) in the E18 vs. D3 contrast. The blue arrows directed at three lipogenic genes (*FASN PLIN2* and *SCD*) predict inhibition by RORA. However, *RORA* and five direct targets (*IGFBP1, LPIN2, PPARGC1A* and *AKR1D1*) are up-regulated in the E18 vs. D3 contrast as indicated by red gene symbols. Further, Ingenuity predicts that *NR1H4* would be slightly activated, which would lead to activation (orange arrows) of four target genes (*CETP, LCAT, LIPC* and *PPARA*), although seven target genes are actually expressed at higher levels in liver of E18 embryos

The contrast of E18 embryos vs. D3 hatchlings revealed another gene network of six transcription factors (*RORA, SMARCD3, ESRRG, NR1H4, PPARGC1A* and S*REBF2*) and their interactions with direct gene targets that control “Lipid Metabolism” (Fig. 9a). Only *SREBF2* was expressed higher in D3 hatchlings, where most of its target genes are involved in lipid metabolism. The RAR-related orphan receptor A (*RORA;* Fig. 9b) was predicted by IPA to be inhibited (blue symbol) and three target genes with blue arrows would be inhibited (down-regulated; green symbols) when compared to E18 embryos, which possessed only five up-regulated target genes (*IGFBP1, LPIN2, NTRK2, PPARGC1A* and *AKR1D1*). Ingenuity predicts that NR1H4 would be slightly activated mainly due to presence of seven DEGs (*CETP, FABP5, LCAT, LIPC, NOS2, PPARA* and *PPARGC1A*) that were highly expressed in liver of the E18 embryos. Ingenuity also predicts that upregulated *NR1H4* would inhibit lipid synthesis (i.e., *ACACA, CYP7A1* and *FASN*).

**Fig. 10:**
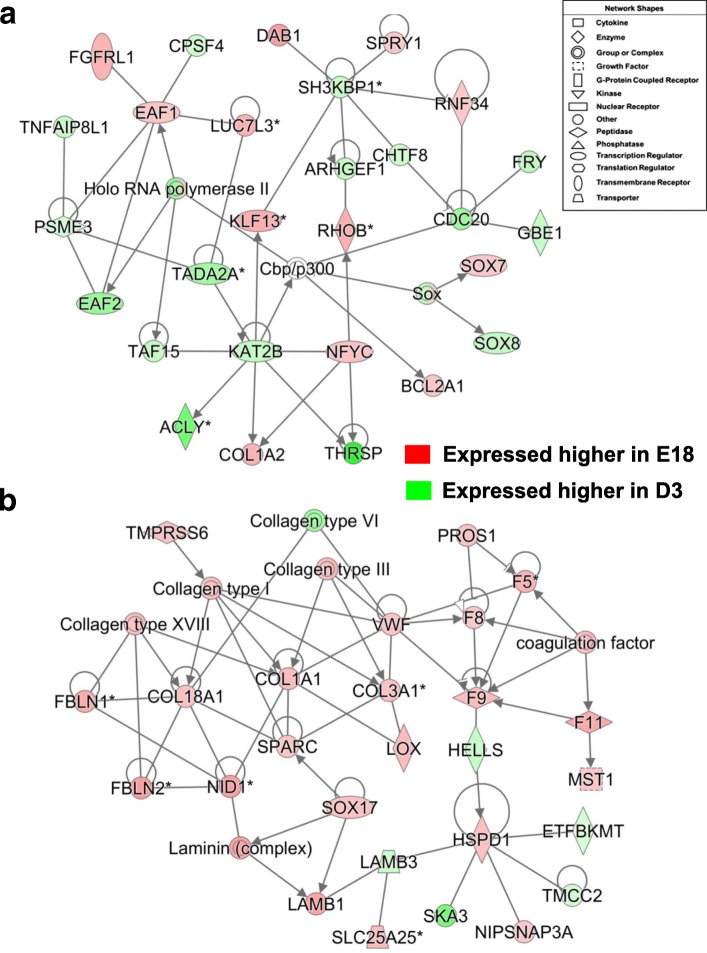
The top panel (**a**) depicts another gene interaction network identified by IPA in the E18 vs. D3 contrast. This gene network, functionally annotated by IPA as “Cell Signaling”, shows interactions of eight transcription factors (*NFYC, THRSP, KAT2B, KLF13, EAF1, EAF2, SOX7* and *SOX8*) and their direct target genes. The second gene network (**b**) was functionally annotated by IPA as “Hematological System Development and Function”. This network of genes, which are highly expressed in liver of E18 embryos, involves interactions among six blood clotting factors [*F5, F8, F9, F11, PROS1* and von Willebrand factor (*VWF*)] with macrophage stimulating 1 (*MST1*), the chromatin remodeling gene [helicase lymphoid specific (*HELLS*)], a mitochondrial chaperone [heat shock protein family D (Hsp60) member 1 (*HSPD1*)], SRY-box 7 (*SOX7*), several collagen genes (*COL1A1*, *COL3A1* and *COL18A1*), fibulin 1–2 (FBLN1, FIBLN2) and components of the extracellular matrix [secreted protein acidic and cysteine rich (*SPARC*), laminin subunit beta-1, − 3 (*LAMB1, LAMB3*) and lysyl oxidase (*LOX*)]

Another gene interaction network from the E18 vs, D3 contrast (Fig. 10a) was functionally annotated by IPA as involved in “Cell Signaling” and under control of eight interacting transcription factors (*THRSP, NFYC, KAT2B, KLF13, EAF1, EAF2, SOX7* and *SOX8*). The lipogenic transcription factor *THRSPA* has direct interactions with two other transcription regulators (*NFYC* and *KAT2B*). In all, 12 genes in this network were expressed higher in E18 embryos, whereas 18 AR-DEGs were over-expressed in liver of D3 hatchlings. The second gene network (Fig. 10b) involves interactions among six clotting factors (*F5, F8, F9, F11, PROS1* and *VWF*), the growth factor gene *MST1*, collagen genes (*COL1A1, COL3A1*and *COL18A1*) and components of the extracellular matrix (*FBLN1–2*, *LAMB1, LAMB3*, *LOX, SPARC* and *SOX17*). This connection of the extracellular matrix with F9 and other coagulation factors is mediated by the direct interaction between two enzymes (*HELLS* and *HSPD1*). Interestingly, most of the AR-DEGs in this network were also expressed higher in liver of E18 embryos than D3 hatchlings. Furthermore, 12 coagulation factors (AR-DEGs) belonging to the canonical “Intrinsic Prothrombin Activation Pathway” were up-regulated in liver of E18 embryos (see Additional file 6).

#### E20 vs. D3 contrast

The E20 vs. D3 contrast identified 1775 AR-DEGs, whose functional analysis is summarized in Table 6. The top canonical pathways occupied by these AR-DEGs were related to “Cholesterol Biosynthesis”, the “Complement System”, “LXR/RXR Activation” and “Intrinsic Prothrombin Activation Pathway”. The top transcription regulators in the E20 vs. D3 contrast were TP53, PPARA, E2F4, E2F1 and FOXO3. Within the “Molecular and Cellular Functions” category, IPA identified “Cell Cycle’, “Cellular Assembly and Organization”, DNA Replication, Recombination, and Repair”, “Lipid Metabolism”, and “Small Molecule Biochemistry” as over-represented sub-categories. Over-populated physiological functions subcategories included “Organismal Survival”, “Connective Tissue Development” and “Tissue Morphology”. The top “Toxicity Functions” identified by IPA included “LXR/RXR Activation, Cholesterol Biosynthesis, Fatty Acid Metabolism” and “FXR/RXR Activation”. Among the top 10 “Up-regulated Genes” in the E20 vs. D3 contrast were *MOGAT1, PDK4, CPT1A* (a major regulator of fatty acid β-oxidation) and *IGFBP2*. The highest DEGs found in the liver of D3 hatchlings are involved in lipogenesis [*THRSPA, SQLE, FASN, SCD, ELOVL5* and *FADS2*).

**Table 6:** IPA summary of the liver transcriptome in the E20 vs. D3 contrast

Top Canonical Pathways	*p*-value	Overlap	Ratio
Super-pathway of Cholesterol Biosynthesis	1.97E-09	69.6%	16/23
Cholesterol Biosynthesis I-III	1.82E-07	90.0%	9/10
Complement System	5.41E-07	58.3%	14/24
LXR/RXR Activation	7.87E-06	34.7%	25/72
Intrinsic Prothrombin Activation Pathway	2.00E-05	55%	12/20
Top Upstream Regulators	*p*-value of overlap		# Target genes
TP53	2.52E-23		257
PPARA	1.23E-20		100
E2F4	3.03E-20		71
E2F1	5.46E-18		105
FOXO3	6.20E-16		77
Top Molecular and Cellular Functions	*p*-value		# Genes
Cell Cycle	8.31E-04 - 5.90E-14		392
Cellular Assembly and Organization	8.35E-04 - 5.90E-14		188
DNA Replication, Recombination, and Repair	8.31E-04 - 5.90E-14		294
Lipid Metabolism	8.39E-04 - 1.88E-11		333
Small Molecule Biochemistry	8.39E-04 - 1.88E-11		382
Physiological System Development and Function	*p*-value		# Genes
Organismal Survival	2.88E-04 - 1.37E-05		470
Connective Tissue Development and Function	2.59E-04 - 1.65E-05		152
Tissue Morphology	2.88E-04 - 1.65E-05		219
Reproductive System Development and Function	8.08E-04 - 4.69E-05		10
Embryonic Development	5.07E-04 - 7.98E-05		35
Top Toxicity Functions	*p*-value	Overlap	Ratio
LXR/RXR Activation	1.04E-05	34.2%	25/73
Cholesterol Biosynthesis	4.67E-05	60.0%	9/15
Fatty Acid Metabolism	5.58E-05	33.3%	22/66
Cell Cycle: G2/M DNA Damage Checkpoint Regulation	6.76E-05	39.0%	16/41
FXR/RXR Activation	1.38E-04	30.4%	24/79
Top Up-regulated Genes	log2 Ratio	Top Down-regulated Genes	log2 Ratio
MOGAT1	5.27	THRSPA	−9.13
PDK4	5.04	CDO1	−6.73
ITGBL1	4.50	SQLE	−6.70
CPT1A	4.27	NSDHL	−6.62
PPM1K	4.16	FASN	−5.84
DNAJC12	3.91	SCD	−5.80
IGFBP2	3.68	ELOVL5	−5.51
CPED1	3.66	FADS2	−5.46
ZBTB16	3.37	CDKN2B	−5.01
MT3	3.36	CYP7A1	−4.98

Ingenuity Pathway Analysis (IPA) was used for functional analysis of 1775 AR-DEGs identified in the E20 vs. D3 contrast (E20/D3 ratio). AR-DEGs with positive log2 ratios are expressed higher in liver of E20 embryos, while negative log2 ratios indicate higher expression in D3 embryos

#### D1 vs D9 contrast

Finally, the contrast of D1 vs. D9 hatchlings yielded 1036 AR-DEGs (Table 7; see Additional file 7). The top canonical pathways identified by IPA were related to cholesterol and stearate biosynthesis, the “Complement System” and “LXR/RXR Activation”. The top upstream regulators and their direct target genes found by IPA in the D1 vs. D9 contrast were PPARA, TP53, E2F4, sterol response element binding factor-2 (SREBF2) and TCF3. Under the “Molecular and Cellular Functions” category, the largest numbers of AR-DEGs were assigned to the “Cell Cycle”, “Lipid Metabolism” and “Small Molecule Biochemistry” subcategories. Under the “Physiological Systems” category, the largest number of AR-DEGs in the D1 vs. D9 contrast were found under “Connective Tissue Development and Function” and Tissue Morphology. The top five “Toxicity Functions” were “Fatty Acid Metabolism”, “Cholesterol Biosynthesis”, “LXR/RXR Activation”, “Cell Cycle: G2/M DNA Damage Checkpoint Regulation” and “Liver Necrosis/Cell Death”. The “Lipid Synthesis” subcategory contained 134 AR-DEGs, 53 were up-regulated in fasted D1 hatchlings and 81 genes were expressed higher in liver of fed D9 hatchlings (see Additional file 7). Three over-represented canonical pathways were related to AR-DEGs controlling “RXR Function”; these were “LPS/IL1 Mediated Inhibition of RXR Function”, “LXR-RXR Activation”, “FXR-RXR Activation”, and “TR-RXR Activation”. Among the highest AR-DEGs found in the liver of fasted D1 hatchlings were *IGFBP2, PDK4, PTGDS*, *PPARGC1A, CPT1A, LPL* and *DIO3* (Table 7). In contrast, the fully-fed D9 hatchling chicks showed higher expression of *CDKN2B* and several lipogenic genes including *SQLE, THRSPA, FADS2, SCD, LSS* and *PNPLA3*.

**Table 7:** IPA summary of the liver transcriptome in the D1 vs. D9 contrast

Top Canonical Pathways	*p*-value	Overlap	Ratio
Superpathway of Cholesterol Biosynthesis	2.07E-08	73.9%	17/23
Cholesterol Biosynthesis I-III	3.03E-06	90.0%	9/10
Complement System	8.89E-06	41.7%	10/24
Stearate Biosynthesis	1.36E-05	36.7%	11/30
LXR/RXR Activation	1.42E-05	25%	18/72
Top Upstream Regulators	*p*-value of overlap		# Target genes
PPARA	4.31E-29		86
TP53	1.01E-24		179
E2F4	8.06E-18		51
SREBF2	8.28E-18		29
TCF3	9.21E-17		59
Top Molecular and Cellular Functions	*p*-value		# Genes
Cell Cycle	5.90E-04 - 5.96E-16		236
Cellular Assembly and Organization	6.09E-04 - 5.96E-16		103
DNA Replication, Recombination, and Repair	4.27E-04 - 5.96E-16		128
Lipid Metabolism	5.51E-04 - 1.94E-15		248
Small Molecule Biochemistry	5.51E-04 - 1.94E-15		281
Physiological System Development and Function	*p*-value		# Genes
Connective Tissue Development and Function	5.90E-04 - 2.82E-07		129
Tissue Morphology	5.90E-04 - 2.82E-07		172
Reproductive System Development and Function	5.51E-04 - 7.32E-07		16
Digestive System Development and Function	5.51E-04 - 1.50E-05		56
Hepatic System Development and Function	8.31E-05 - 1.50E-05		55
Top Toxicity Functions	*p*-value	Overlap	Ratio
Fatty Acid Metabolism	1.66E-07	30.3%	20/66
Cholesterol Biosynthesis	5.16E-07	60.0%	9/15
LXR/RXR Activation	1.74E-05	24.7%	18/73
Cell Cycle: G2/M DNA Damage Checkpoint Regulation	7.32E-05	29.3%	12/41
Liver Necrosis/Cell Death	1.07E-04	15.9%	35/220
Top Up-regulated Genes	log2 Ratio	Top Down-regulated Genes	log2 Ratio
CHODL	3.79	CDKN2B	−8.46
SRL	3.78	SQLE	−7.71
IGFBP2	3.05	THRSPA	−6.39
PDK4	2.95	FADS2	−6.19
PTGDS	2.89	SCD	−6.11
ACBD7	2.88	CDO1	−6.10
PPARGC1A	2.80	UCHL1	−5.86
CPT1A	2.77	LSS	−5.40
LPL	2.73	ENPEP	−5.17
DIO3	2.73	PNPLA3	−5.14

Ingenuity Pathway Analysis (IPA) was used for functional analysis of 1036 AR-DEGs identified in the D1 vs. D9 contrast (D1/D9 ratio). AR-DEGs with positive log2 ratios are expressed higher in liver of D1 embryos, while negative log2 ratios indicate higher expression in D9 embryos

**Fig. 11:**
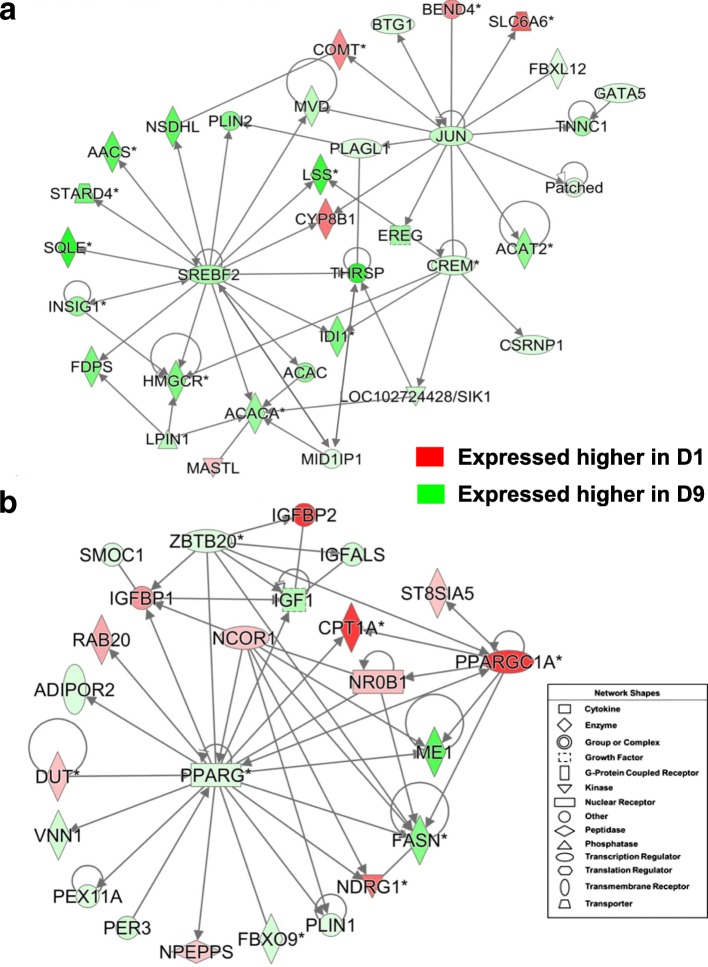
The D1 vs. D9 contrast revealed two distinct gene networks centered on interaction among multiple lipogenic transcription factors. The gene network shown in Panel **a** was functionally annotated as “Lipid Metabolism”. Seven upstream regulators [*THRSP,* MID1 interacting protein 1 (*MID1IP1* or *THRSPL*)*, SREBF2,* cAMP responsive element modulator (*CREM*)*,* Jun proto-oncogene, AP-1 transcription factor subunit (*JUN*)*,* BTG anti-proliferation factor 2 (*BTG2*) and PLAG1 like zinc finger 1 (*PLAGL1*] and their direct target genes were mainly expressed at higher levels in liver of fed D9 hatchlings. The gene network in Panel **b** focusses on interactions of *PPARG*, which was expressed higher in liver of fully-fed D9 hatchlings, with four other transcription factors, three of which (*NCOR1, NR0B1* and *PPARGC1A*) were more abundant in liver of fasted D1 hatchling chicks

Two gene interaction networks from the D1 vs. D9 contrast were functionally annotated by IPA as involved in “Lipid Metabolism” (Fig. 11). As shown in Fig. 11a, several lipogenic transcription factors [*SREBF2, THRSPA, MID1IP1* (a THRSP paralog)*, PLAGL, JUN* and *CREM*] control expression of multiple metabolic enzymes (*ACACA, HMGCR, FDPS, SQLE, AACS, LSS, CYP8B1*, *IDI1, MVD, COMT, FBXL12* and *ACAT2*), kinases (*MASTL, SIK1*), a phosphatase (*LPIN1*), and transporters (*STARD4* and *SLC6A6*). The gene network in Fig. 11b shows interactions of five transcription factors (*PPARG, PPARGC1A, NR0B1, NCOR1*and *ZBTB20*) with key lipogenic metabolic enzymes (*FASN, ME1, CPT1A*), growth factors (*IGF1*), binding proteins (*IGFBP1, IGFBP2, IGFALS*) and the adiponectin receptor (*ADIPOR2*). Other direct targets of *PPARG* shown in this gene network were *NDRG1, PLIN2, PER3* and *VNN1*.

**Fig. 12:**
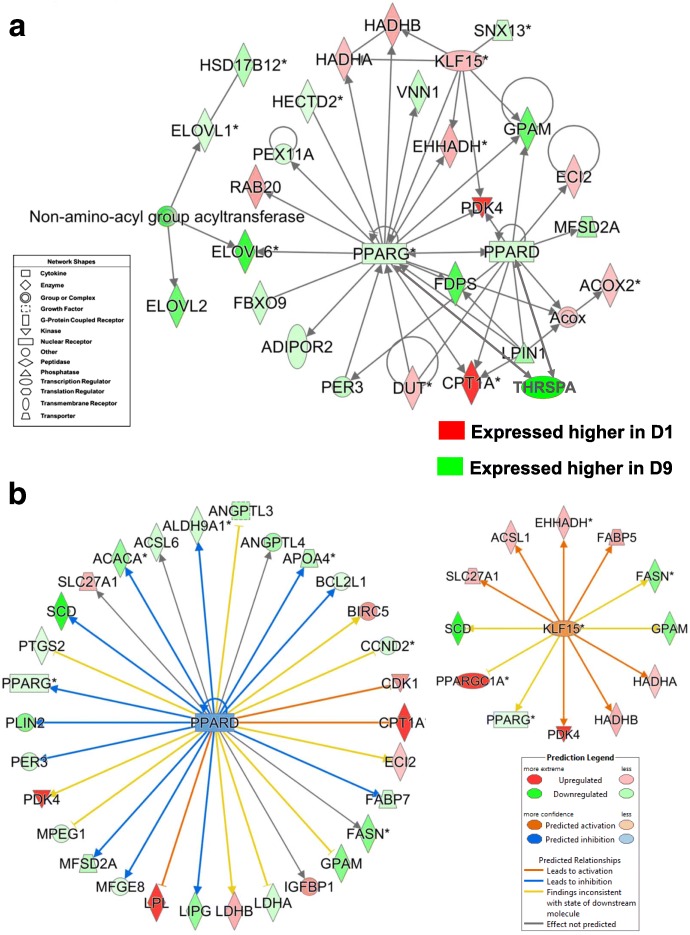
This gene network from the D1 vs. D9 contrast was functionally annotated by IPA as “Energy Production, Lipid Metabolism”. Panel **a** shows interactions among several lipogenic transcription factors [*THRSPA, PPARG, PPARD* and Kruppel like factor 15 (*KLF15*)] and their direct target genes, which are mainly expressed at greater levels in liver of D9 hatchlings. However, four target genes of *KLF15,* itself upregulated, were up-regulated in the liver of D1 hatchlings [i.e., hydroxyacyl-CoA dehydrogenase trifunctional multi-enzyme complex subunit alpha (*HADHA*), *HADHB*, enoyl-CoA hydratase and 3-hydroxyacyl CoA dehydrogenase (*EHHADH*) and pyruvate dehydrogenase kinase 4 (*PDK4*)]. Panel **b** shows known direct targets of *PPARD* and *KLF15* and Ingenuity predictions of inhibition (blue arrows) or activation (dark orange arrows) of both the up-stream regulator and its respective gene targets. Ingenuity predicts inhibition of *PPARD*, due to the majority of downregulated target genes in the D1 vs. D contrast, which would lead to inhibition of 12 genes (blue arrows), whereas the blunt orange lines predict that PPARD actively inhibits cyclin dependent kinase 1 (*CDK1*), carnitine palmitoyltransferase 1A (*CPT1A*) and lipoprotein lipase (LPL), which are highly expressed in liver of fasted D1 hatchlings. Ingenuity predicts that activation of *KLF15* in fasted D1 hatchlings would lead to activation of seven known target genes [solute carrier family 27 member 1 (*SLC27A1*), acyl-CoA synthetase long chain family member 1 (*ACSL1*), *EHHADH, FABP5, HADHA, HADHB* and *PDK4*], although uncertainty exists about KLF15’s action on five other AR-DEGs as indicated by the blunt yellow edges

The final gene interaction network (Fig. 12a) was also identified in the D1 vs. D9 contrast and functionally annotated by IPA as belonging to “Energy Production” and “Lipid Metabolism”. This network is centered on interactions among four lipogenic TFs (*THRSPA, PPARG, PPARD* and *KLF15*), where *PPARG* and *PPARD* share seven target genes (*PER3, DUT, CPT1A, THRSPA, LPIN1, ACOX2* and *PDK4*). The TF *PPARG* and several of its direct targets are expressed at higher levels in liver of D9 hatchlings, including *ADIPOR2, FBXO9, ELOVL6, ELOVL2, ELOVL1, HSD17B12, PEX11A, HECTD2, VNN1* and *FDPS.* The fasting-induced transcriptional factor *KLF15* and four direct gene targets (*HADHA, HADHB, EHHADH* and *PDK4*) are expressed higher in liver of fasted D1 hatchlings. The Ingenuity Upstream Regulator Analysis (Fig. 12b) identified 30 AR-DEGs in the D1 vs. D9 contrast as direct targets of *PPARG,* where only 9 genes were up-regulated in the fasted D1 hatchlings. Consequentially, IPA predicted that PPARG would be inhibited (blue gene symbol and blue arrows), since 21 of its target genes are down-regulated (green symbols) AR-DEGs in this contrast. Based on known relationships in the Ingenuity Knowledge Base, Ingenuity predicts that inhibited PPARG would block the expected activation (blunt orange lines) of *CDK1, CPT1A* and *LPL*. Ingenuity predicts that *KLF15* would be activated, which would lead to activation (up-regulation) of seven direct targets (*ACSL1, EHHADH, FABP5, HADHA, HADHB* and *PDK4*) as indicated by the orange arrows. Interestingly, *KLF15*, a key transcriptional regulator of gluconeogenesis, was highly expressed in liver of fasted D1 hatchling chicks, as revealed by three pairwise contrasts (E18 vs. D1; D1 vs. D3; and D1 vs. D9).

### qRT-PCR analysis and verification of differential expression of 15 candidate DEGs

**Fig. 13:**
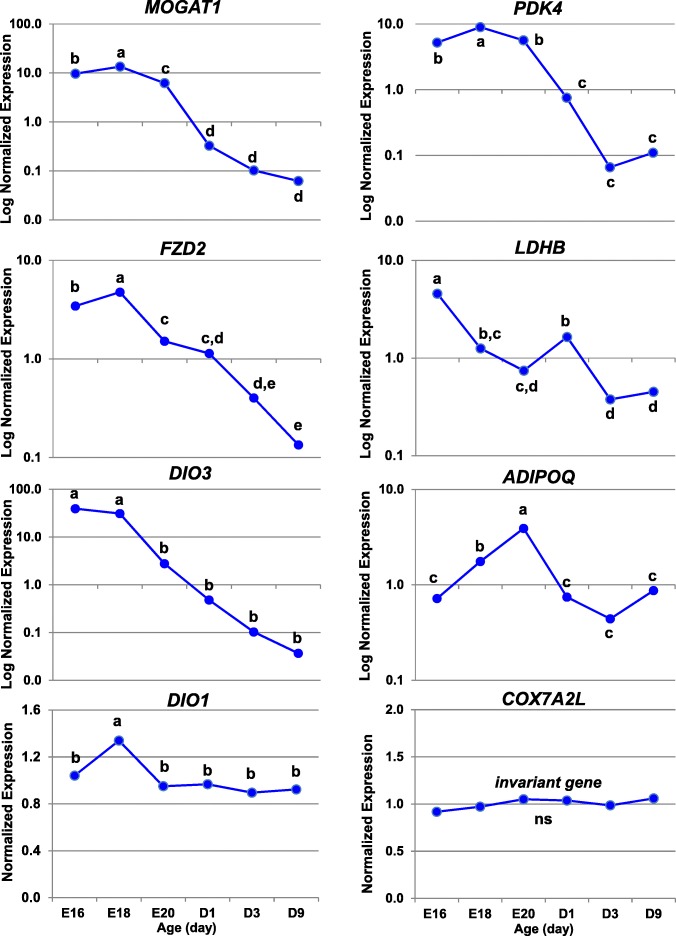
This figure depicts the qRT-PCR analysis of seven “lipogenic” DEGs identified by genome array analysis. Each value represents the mean ± SEM of four biological samples (individual embryo or hatchling liver) and their two technical replicates in the qRT-PCR analysis. Values possessing different superscripts are significantly different as determined by ANOVA and mean separation using Duncan’s Multiple Range Test in SAS. These genes are mainly involved in lipolysis and energy catabolism; and all are expressed at higher levels in embryos, with the exception of the invariant gene cytochrome c oxidase subunit 7A2 like *(COX7A2L)*. The invariant gene was one of three housekeeping genes used for normalization of transcript abundance in liver of embryos and hatchling chicks. *Gene symbols*: Monoacylglycerol O-acyltransferase 1(*MOGAT1*); pyruvate dehydrogenase kinase 4 (*PDK4)*; frizzled class receptor 2 (*FZD2*); lactate dehydrogenase B (*LDHB*); iodothyronine deiodinase 3 (*DIO3*); iodothyronine deiodinase 1 (*DIO*); and adiponectin, C1Q and collagen domain containing (*ADIPOQ*)

**Fig. 14:**
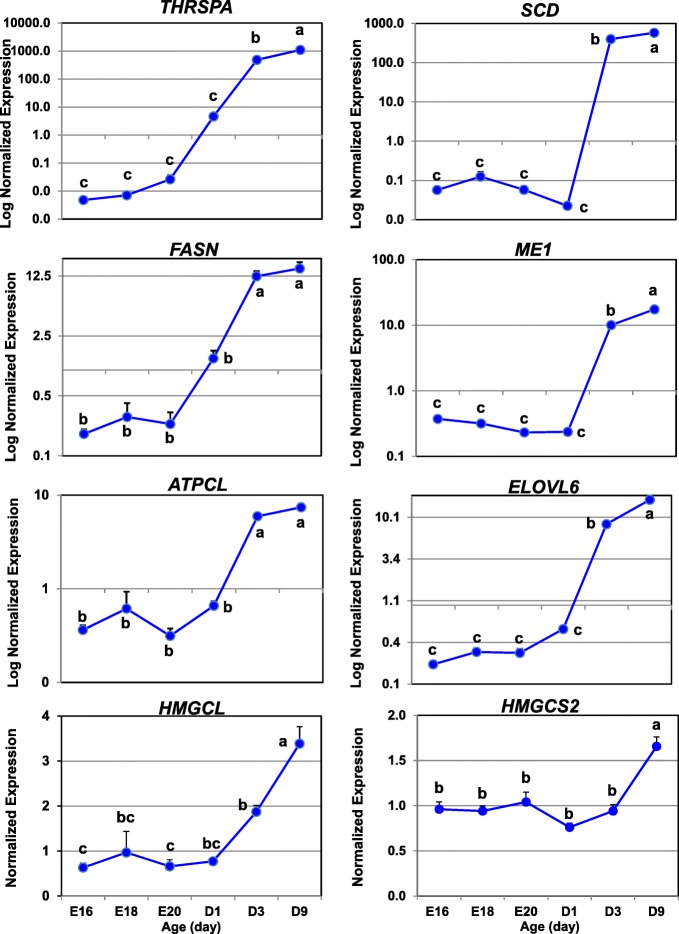
This figure shows the qRT-PCR analysis of eight “lipogenic” genes, which were originally identified as DEGs by genome array analysis. These genes were more abundant in liver of hatchlings. Each value represents the mean ± SEM of four biological samples (individual embryo or hatchling liver) and their two technical replicates. Values possessing different superscripts across ages were significantly different as determined by ANOVA and mean separation using Duncan’s Multiple Range Test in Statistical Analysis System (SAS)*Gene symbols*: Thyroid hormone-responsive Spot 14 protein, alpha (*THRSPA*); delta-9 desaturase (*SCD*); fatty acid synthase (*FASN*); malic enzyme 1, NADP(+)-dependent, cytosolic (*ME1*); ATP citrate lyase (*ATPCL*); ELOVL fatty acid elongase 6 (*ELOVL6*); 3-hydroxy-3-methylglutaryl-CoA lyase (*HMGCL*); and 3-hydroxy-3-methylglutaryl-CoA synthase (*HMGCS2*)

Hepatic expression patterns of seven DEGs that were expressed higher in embryos and an invariant gene (*COX7A2L*) during the embryo-to-hatchling transition were examined by qRT-PCR analysis (Fig. 13). Six genes (*MOGAT1, PDK4, FZD2, LDHB, DIO3* and *ADIPOQ*) show higher log-based expression in embryos and a sharp decline after hatching. Surprisingly, hepatic expression of *DIO1* was relatively stable, except in E18 embryos where *DIO1* was slightly elevated. The invariant gene (*COX7A2L*) was used by geNorm software for normalization of the qRT-PCR expression levels. The expression patterns of eight lipogenic genes during the embryo-to-hatching transition are depicted in Fig. 14. The hepatic expression of *THRSPA* and *SCD* was log-scale and increased exponentially, reaching a plateau in liver of fed hatchlings between D3 and D9. Similar log-scale expression patterns were found for fiv other metabolic genes (*ME1, SCD, FASN, ATPCL* and *ELOVL6*), while *HMGCL* and *HMGCS2* only showed a slight increase in transcript abundance in D9 hatchlings.

**Fig. 15:**
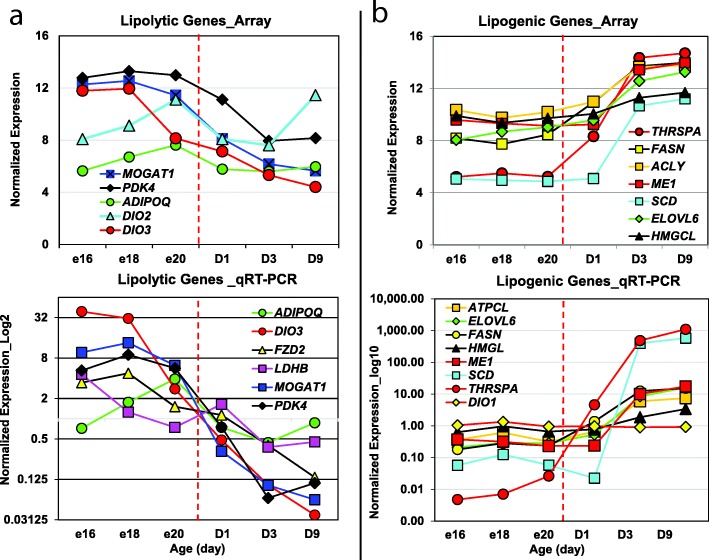
This figure provides the side-by-side comparison of gene expression levels determined by genome microarray analysis and their verification by qRT-PCR analyses for 15 candidate DEGs. This four panel figure provides visual contrasts of lipolytic (Panel **a**) and lipogenic (Panel **b**) genes and their differential expression determined by two independent analyses of 24 biological samples (12 embryos and 12 hatchlings). In Panel **a**, the normalized expression levels of five lipolytic DEGs from genome array analysis and time-course verification by qRT-PCR analysis are displayed in respective order, with genome array data in the top panel and its verification by qRT-PCR analysis in the bottom panel. Panel **b** shows hepatic expression patterns of seven lipogenic genes (*THRSPA, SCD, ME1, FASN, ELOVL6, ACLY* and *HMGCL*) as determined by genome array (top) and by qRT-PCR (bottom) analyses. Log scale expression levels were achieved by qRT-PCR analysis as shown in both bottom panels. Relative expression levels obtained by these two independent methods were used for Pearson’s correlation analysis and the determination of the Pearson’s correlation coefficient (see Additional file 8). *Note*: *DIO1* was considered an invariant gene; therefore, *DIO1* expression was not included in the Pearson’s Correlation Analysis

Figure 15 provides a side-by-side comparison of genome array analysis vs. qRT-PCR analysis for 13 “candidate” genes across 6 developmental ages (E16-D9). In general, transcript abundance determined by genome array analysis was of a lower magnitude than the dynamic log-scale range provided by qRT-PCR analysis, although patterns of gene expression were similar between thetwo analytical methods. Three lipolytic DEGs identified by genome array analysis (Fig. 15a, top) exhibited higher expression in liver of embryos than in hatchings [i.e., *DIO3, MOGAT1* and pyruvate dehydrogenase kinase 4 (*PDK4*)]. The deactivating deiodinase *DIO2* and the adipokine *ADIPOQ* showed a progressive increase in hepatic expression from E16 to a peak in E20 embryos. Although *DIO2* levels were lower in D1 and D3 hatchlings, a peak in abundance was reached in D9 hatchlings. The qRT-PCR analysis (Fig. 15a, bottom) revealed high magnitude (log2) expression of *DIO3, MOGAT1* and *PDK4,* which were highest in E16 and E18 embryos and lowest in liver of fully-fed D3 and D9 hatchlings. Likewise, the hepatic abundance of *ADIPOQ, FZD2* and *LDHB* transcripts were also elevated in embryos compared to hatchlings, albeit with lower amplitudes. Seven DEGs identified by genome array analysis (Fig. 15b, top) displayed relatively low expression in liver of embryos with a sharp rise in expression in fully-fed D3 and D9 hatchlings. The developmental pattern and large-amplitude expression of *THRSPA*, *ME1* and *ELOVL6* transcripts were almost identical and reached a plateau in liver of fed D3 and D9 hatchlings. *HMGCL* levels were similar across embryonic ages and only slightly greater in D3 and D9 chicks. Also, the abundance of *SCD* was low in embryos and fasted D1 hatchlings, with a sharp increase in fed D3 and D9 hatchlings. In contrast, qRT-PCR analysis (Fig. 15b, bottom panel) shows an extremely high abundance of *THRSPA* and *SCD* in liver of D3 and D9 hatchlings that was several orders of magnitude greater than in liver of embryos (E16-E20). Five hepatic genes (*FASN, ME1, ATPCL, ELOVL6* and *HMGCL*) had higher expression levels, albeit with a lower amplitude, in D3 and D9 hatchlings. We were surprised to find that hepatic expression of *DIO1* was rather stable across embryonic (E16-E20) and hatchling (D1-D9) ages. Furthermore, *DIO1* was not detected as a DEG by genome array analysis or qRT-PCR analysis; therefore, *DIO1* was considered an invariant gene and not included in the Pearson’s Correlation Analysis.

The Pearson’s correlation coefficient (*r* = 0.929; 11 degrees of freedom) indicates a highly significant (*P* ≤ 0.01) correlation between gene expression levels obtained from both microarray and qRT-PCR analyses (Additional file 8). As expected, there was close agreement in hepatic transcript abundance between expression platforms (microarray and qRT-PCR analyses); the major difference was the large-magnitude log-scale expression levels achieved by qRT-PCR analysis, albeit the developmental expression patterns were quite similar.

## Discussion

The present study of liver during the embryo-to-hatchling transition (or peri-hatch period) provides a detailed view of the innate choreography of major transcriptional responses involved in the abrupt switch from a lipid-laden ectotherm (embryo) to a free-living endotherm (hatchling chick). We have identified several transcription factors (*PPARA*, *PPARGC1A, KLF15, SIRT1, SERTAD2* and *NR1H4*) which interact with each other and their respective target genes in late embryos as they utilize yolk lipids and prepare for hatching on E21. Upon feeding, hatchling chicks abruptly increase expression of several ligand-activated transcription factors (*THRSPA, SREBF2, PPARG, PPARD* and *KLF11*) which in turn control numerous down-stream metabolic enzymes, transporters and kinases/phosphatases to support greater rates of energy expenditure for lipogenesis, adipogenesis and somatic growth. Hierarchical clustering revealed two opposing patterns of gene expression in liver during the peri-hatch period, where Cluster A had higher expression in embryos, which sharply declined after hatching (see Fig. 1). In contrast, Cluster B genes exhibited lower expression in embryos, which was followed by an exponential increase in abundance of numerous lipogenic genes in fully-fed D3 and D9 hatchlings. These two distinct patterns represent clusters of functionally-related genes that are similar to those found in our original transcriptional profiling of liver in embryos and hatchlings during the peri-hatch period using a prototype 3.2 K chicken liver microarray [19, 30, 31]. Self-organizing maps (SOMs) analysis of the first transcriptional scans using our 3.2 K liver cDNA array (NCBI GEO Platform No. GPL1742) revealed similar distinct patterns of transcript abundance. Cluster 11 contained 46 genes with higher expression in embryos, whereas Cluster 3 (21 genes) had lower expression in embryos with a sharp elevation in hatchlings [19]. Higher resolution of functionally related gene clusters, unique to embryos and to hatchling chicks, was achieved with our spanning-tree clustering method (see Fig. 3 in [31]). Our original transcriptional profiling analysis with a low density 3.2 K microarray provided us with a coarse view of the topography of hepatic gene expression during the embryo-to-hatchling transition. The present transcriptional analysis using chicken genome arrays has greatly expanded the repertoire of responsive hepatic genes involved in the embryo-to-hatchling transition and considerable insight into the regulatory and metabolic pathways that control the abrupt switch from embryonic ectotherm to hatchling endotherm.

### Transcriptional regulation of metabolism during the peri-hatch period

The present study utilized multiple pairwise contrasts between embryos and hatchlings to gain even greater resolution by populating canonical pathways and biological processes with DEGs, which provide homeorhetic control over metabolism during the peri-hatch period. The primary contrast of all embryos against all hatchlings revealed a large number of hepatic DEGs (284 genes) that are involved in lipid metabolism (Table 1). And of these, 149 DEGs belong to the lipid synthesis pathway, where only one-third (54) of the DEGs are more abundant in liver of embryos. We discovered 14 up-stream regulators that control transcription of a large number of metabolic enzymes, transporters, phosphatases and kinases in liver of embryos and hatchlings (see Fig. 4). Membrane-bound and nuclear ligand-activated receptors and G-protein coupled receptors (GCPRs) fine tune and amplify the signals mediated by a few critical transcription factors. These enhanced metabolic activities are enabled by a robust thyroid axis and deiodinases with opposing actions. In the embryo liver, DIO3 converts the prohormone T_4_ into inactive rT_3_ until the moment the embryo pips through its shell and begins pulmonary respiration [16]. Furthermore, the abrupt rise in regulated body temperature (Tb = 42–44 °C) of precocial hatchling chicks is supported by increased hepatic DIO2 activity, which converts T_4_ into metabolic T_3_, the major hormone of thermoregulation and energy expenditure. *DIO3* was one of the highest DEGs in liver of embryos, whereas *DIO2* was the highly-expressed and opposing deiodinase found in liver of hatchlings by multiple contrasts. Initially, DIO1 was considered to be the major hepatic deiodinase providing active thyroid hormone (T_3_) for rapid growth and metabolism in hatchling chicks [15, 18]. The present study clearly shows that *DIO2* expression increases in E20 liver to provide metabolically-active thyroid hormone (T_3_) for hatching and reaches maximum expression in fully-fed rapidly-growing D9 chicks. The importance of DIO3 in deactivating T_4_ was demonstrated by the D1 vs. D9 contrast, where *DIO3* was highly expressed in liver of fasted D1 hatchlings and sharply depressed in fed D3 and D9 hatchings. The opposing actions of *DIO3* and *DIO2* on lipid catabolism or lipogenesis (respectively) were evident across multiple contrasts of embryo and hatchling liver (see Additional file 3).

As expected from our previous transcriptional scans [19, 30–35], major lipogenic transcriptional factors (*THRSPA, SREBP2, PPARG, PPARD* and *KLF11)* interact with each other to control numerous target genes encoding metabolic enzymes, transporters and kinases/phosphatases that support lipogenesis and the thermogenic drive, which sharply increases after hatching and consuming high-energy high-protein feed. Of particular interest, was the over-representation of AR-DEGs (27 genes) observed in the “THR-RXR Activation” pathway (see Additional file 4), where the most abundant DEGs expressed in embryo liver was *DIO3*, which deactivates both prohormone (T_4_) and metabolically-active (T_3_) thyroid hormone, whereas *DIO2* activates the thyroid axis by converting T_4_ to T_3_, the active thyroid hormone and major regulator of energy metabolism and lipogenesis. Furthermore, T_3_ bound to its ligand-activated receptor (THRB) sequentially activates triple T_3_-THRB binding sites on the *THRSP* promoter to provoke an exponential increase in transcription of the *THRSP* gene [36]. In the present study, *THRSPA* followed a distinct sigmoidal expression pattern during the embryo-to-hatchling transition (see Fig. 15b), as demonstrated by qRT-PCR analysis. The increased generation of T_3_ by *DIO2* in liver of hatchlings provides ligand for binding to and activating the THRB, an essential step in the progressive activation of *THRSPA*. Furthermore, activated nuclear receptor (T_3_-THRB) also serves as a binding partner (heterodimer) for several other nuclear receptors (FXR, LXR, RXR and VDR). For example, the THRSP promoter in the rat has three T_3_-THR response elements which act synergistically to provide maximal transcription of the *THRSP* gene [36]. Transcription of THRSP is regulated by several other ligand-activated nuclear receptors: SREBP1c via sterol response elements (SRE) [37], retinoic acid-RXR [38], NR1I3, pregnane X receptor (PXR), CAR-RXR complex [39], estrogen via estrogen response elements (ERE) [40], and several metabolites: [glucose bound to carbohydrate regulatory element binding protein (ChREBP) [41], polyunsaturated fatty acid [42] and prostaglandin [43]. In the present study, we identified a lipogenic gene network in the D1 vs. D9 contrast (see Fig. 11a) where *THRSP* and its paralog MID1 interacting protein 1 (*MID1IP1*) [also known as THRSPL/S14R] were up-regulated in liver of D9 hatchlings with direct interactions with *SREBF2* and *ACACA*, which encodes the rate limiting enzyme in fatty acid synthesis. Apparently, both *THRSP* and *MID1IP1* are glucose-responsive genes, which have overlapping roles in regulating expression of several lipogenic genes [44]. These observations strongly support activation of the thyroid axis in hatchlings to increase energy expenditure to meet demands of endothermy and rapid somatic growth. The present study affirms and extends our original idea that *THRSPA* controls transcription of several metabolic enzymes and transporters, which contribute to lipogenesis and the exponential growth exhibited by newly-hatched (meat-type) chicks. Previously, we have shown abundant expression of *THRSPA* in abdominal fat and liver of the chicken and demonstrated its involvement in adipogenesis and lipogenesis [19, 30, 31, 33, 34, 45, 46]. Furthermore, we discovered a 9 bp insertion/deletion polymorphism in the DNA binding domain, which is associated with the abdominal fatness trait in multiple populations of broiler chickens. Others have shown that mutations in the *THRSPA* gene are associated with fatness phenotypes in chickens [47, 48], ducks [49] and geese [50]. Taken together, *THRSPA* is obviously a major regulatory of lipogenic genes expressed in liver and visceral fat of the chicken and other poultry, like ducks and geese. A recent study in mice provides direct evidence that enhanced *THRSP* expression is controlled via a LXR-mediated, SREBP-dependent mechanism [51]. These extensive findings strongly support the full recognition of *THRSPA* as a principal transcriptional regulator of lipogenesis and adipogenesis in the chicken and other domestic birds.

The transcriptional choreography of the metabolic switch from embryo to hatchling includes interaction of several ligand-activated nuclear receptors [52] namely, farnesoid X receptor (*FXR* or *NR1H4*; activated by bile acids and cholesterol derivatives), liver X receptor (*LXR*; activated by oxysterols and other cholesterol derivatives), the orphan nuclear receptor small heterodimer partner (*SHP* or *NROB2*), which regulates metabolism of cholesterol, bile acids, fatty acids and glucose [53], and *PPARA*. The overlapping roles and interplay of the ligand-activated nuclear receptors LXR and RXR has been elegantly described as a “*ying* and *yang* relationship”, which precisely regulates bile acid, cholesterol and triglyceride metabolism [52]. Presently, most pairwise contrasts of embryo and hatchling liver transcriptomes have identified activation of LXR-RXR, and FXR-RXR heterodimers as over-represented metabolic pathways. Furthermore, we discovered other examples of opposing (or *ying-yang*) transcriptional regulators (i.e., *PPARA* vs. *PPARG*; *SIRT1* vs. *PPARG*; *SIRT1* vs. *SREBF2*; *PPARG* vs. *PPARGC1A*; *SREBF2* vs. *PPARA*; *NR0B2* vs. *NR1H4*; *THRSPA* vs. *PPARA,* and *THRSPA* vs. *PPARGC1A*) and metabolic enzymes (i.e., *DIO3* vs. *DIO2*). Furthermore, we identified 9 of the 15 known members of the Kruppel-like transcription factor (KLF) family [54] as DEGs (*KLF2, KLF3, KLF6, KLF9, KLF10, KLF11, KLF12, KLF13* and *KLF15*) from our 14 pairwise contrasts (Additional file 3). In particular, KLF15 seems to act as a *homeorhetic* transcription factor capable of “switching between lipogenesis and gluconeogenesis during fasting” [55]. This dynamic adaptation in energy metabolism is achieved by KLF15 forming complexes with LXR-RXR heterodimers on the promoter of mouse *SREBF1c*, thereby inhibiting transcription of this major lipogenic transcription factor. The ability of KLFs to either activate or repress gene transcription depending upon metabolic demands was recently reviewed in an article that precisely coined their dynamic action as “crippling and uncrippling metabolic pathways” [54]. The present examination of liver transcriptomes clearly shows interactions, interdependence and interconnectivity of multiple upstream regulators and their direct target genes in regulating lipolysis in late embryos and lipogenesis in fully-fed D3 and D9 hatchling chicks.

The NAD^+^-dependent deacetylase *SIRT1* is involved in alternative energy utilization, like β-oxidation of lipid and gluconeogenesis [56–58], which are critical metabolic processes in liver of late embryos. Furthermore, *SIRT1* enhances thyroid (T_3_) control over expression of several lipogenic genes, including *CPT1A*, *PPARA*, *PPARGC1A, PDK4, PCK1* and *SREBP-1c* [59]. Another transcription factor up-regulated in liver of embryos was prospero-related homeobox 1 (*PROX1*), which is important for hepatic embryogenesis and a negative regulator of triglyceride synthesis via mTOR signaling [60]. Thus, the metabolism of the late chicken embryo is under the intricate control of multiple transcription factors and directed at utilization of yolk lipids that amass in liver. The retained yolk sac provides nutrients and lipid-derived energy until the hatchling eats its first meal and becomes a self-sufficient endotherm. In the present study, we included D1 hatchlings, which had not been fed and still dependent on yolk-lipid and swallowed albumen to reach and maintain endothermy. Full and untethered expression of multiple transcription factors and their target genes that contribute to lipogenesis occurs only after the hatchling chick is fully-fed (D3 and D9) and the residual yolk sac depleted.

Another interesting TF identified in the four contrasts of embryos and hatchlings was SERTA domain containing 2 (*SERTAD2;* also known as *TRIP-Br2*), which was expressed at highest levels (log2 ratio = + 1.75) in the E16 vs. D1 contrast. The differential expression of *SERTAD2* was found in three additional contrasts of embryos against hatchlings (E vs. H, E16 vs. D3 and E18 vs. D1). This newly-discovered transcriptional co-regulator is highly expressed in visceral fat of obese humans; furthermore, *SERTAD2* appears to control lipolysis, oxidative metabolism and thermogenesis in mouse models [61–63]. Knockout of the *SERTAD2* gene in mice prevents diet-induced obesity, insulin resistance and inflammatory responses in visceral fat. Our discovery of up-regulated *SERTAD2* in liver of E16 and E18 chick embryos suggest that this transcriptional co-regulator could contribute to transcriptional control of lipolysis and oxidative metabolism in the lipid-laden liver of late chick embryos via interactions with known lipolytic TFs (i.e., *PPARA, PPARGC1A, SIRT1* and *NR1H4*).

Our present transcriptional study has also revealed acquisition of a competent blood clotting system in E16 and E18 embryos, which exhibit abundant differential expression of multiple coagulation factors and collagen genes (see Figs. 6a and 10b; Additional files 4 and 6). The deacetylase SIRT1 appears to control essential biological pathways like blood coagulation through selective acetylation of clotting factors and the closely related acute-phase response proteins [57]. SIRT1 also enhances PPARA-PPARGC1A driven lipolysis and lipid depletion of adipocytes, which in turn activates gluconeogenesis a vital metabolic response of the embryo. The largest number of coagulation genes was found in the E18 vs. D3 contrast, where 10 clotting factors and 7 collagen genes were expressed higher in the E18 embryo. Similarly, 12 blood clotting factors were found in the E20 vs. D3 contrast, where 9 coagulation and 3 collagen genes were up-regulated in the liver of E20 embryos. In addition, fibrinogen alpha (*FGA*) was over-expressed in E18 and E20 embryos (see Additional file 3). The transcriptional analysis of the chicken yolk sac in late chicken embryos (E13-E21) revealed peak abundance of fibrinogen (β and γ subunits; *FGB* and *FGG*) genes at E19 [8]. Presumably, a competent blood coagulation system is crucial to prevent excessive blood loss in the embryo during absorption of its residual yolk sac into the visceral cavity, destruction of the chorioallantois, and emergence of the E21 embryo as a hatchling chick [64].

### Hepatic expression of the feather keratin gene *FKER*

Interestingly, *FKER* was the most DEG found in liver of E16 embryos (see Additional file 3). In fact, seven distinct transcripts represented on the Affymetrix Chicken Genome Array correspond to feather keratin or feather keratin-like genes (Table 8), which reside at multiple loci on the chicken genome (*GGA1, GGA25* and *GGA27*) [65–67]. Five of these over-expressed *FKER* transcripts map to *GGA*25, while *FK1* (BI064513) resides on *GGA*1 and XM_424568 is located on *GGA*27. At first glance, abundant expression of *FKER* in liver of the E16 embryo seems difficult to explain. However, we originally discovered the exceptional, and very high, abundance of *FKER* in liver of E15 embryos from Leghorn (egg-type) chickens carrying the riboflavin binding-protein deficiency mutation *rd/rd*, with or without riboflavin rescue [35]. We proposed that the abundant hepatic expression of *FKER* exclusively in E15 embryos could be related to massive engorgement of the embryo’s liver with yolk lipids, which normally occurs at this stage of embryonic development. In fact, an early study described the presence of lipids in the embryo’s developing cutaneous feather, which was likely associated with “pre-keratin”, where lipids function as the scaffold for feather keratin formation [68]. In the mouse, cytoskeletal keratin 8 (*Krt8*) is required for structure and integrity of hepatocytes [69]. Furthermore, ablation of the *Krt8* in mice results in oxidative stress and excessive accumulation of lipid and protein metabolites [70]. Examination of the pigeon’s lactating crop transcriptome has revealed up-regulation of several β-keratin genes, including *FKER*, which are ultimately involved in production of lipid-laden crop milk [71]. The unique synthesis of crop milk in *Columbiformes* involves prolactin-mediated cornification and lipid synthesis of crop epithelial cells, which are desquamated and sloughed off to form “crop milk”, which is fed to the altricial squabs. This transcriptional study of pigeon crop milk production clearly shows over-expression of multiple β-keratin genes, which are associated with de novo lipid synthesis and cornification of crop epithelium. Of particular interest, all of the differentially-expressed lipogenic genes found in the lactating pigeon crop sac were identified as DEGs in the present study, including the highly-expressed lipogenic transcription factor *THRSPA* [71]. The present descriptive study of the liver transcriptome during the embryo-to-hatchling transition validates our original discovery of abrupt over-expression of *FKER* in E15 Leghorn (egg-type) embryos [35], which coincides with massive accumulation of yolk lipid in liver that fuels the final embryonic growth phase and emergence of the hatchling chick. Additional support for the ectopic expression of hepatic *FKER* transcripts in E16 Leghorn embryos is provided by our initial observation of differential expression of a cDNA target (pgf1n.pk001.j5; GenBank BI064513) on a low-density 3.2 K chicken microarray (NCBI GEO Platform GPL1742) [19]. This cDNA clone (pgf1n.pk001.j5) was sequenced from a normalized adipose cDNA library derived from embryo and hatchling chickens. Furthermore, our recent transcriptional study of liver from *rd/rd* Leghorn embryos, riboflavin-deficient and riboflavin rescued, utilized the Arizona 20.7 K chicken oligo array (NCBI GEO Platform GPL6049), where three 70-mer oligo targets [Roslin Institute *Gallus gallus* (RIGG) oligo; RIGG10897, RIGG14163 and RIGG14953] had 19-times greater abundance in liver of riboflavin-rescued embryos than riboflavin-deficient embryos between E13 and E15. Thus, three independent chicken microarray platforms and multiple expressed-sequence tag (EST) sequences in GenBank clearly support abundant “ectopic” expression of *FKER* in liver of E15 and E16 embryos from both egg-type (Leghorn) [35] and, presently, meat-type (broiler) chicken breeds. However, the biological function of *FKER* in the lipid-laden liver of late embryos and the relationship between hepatic expression and cutaneous expression of *FKER* in embryos will require further definitive study.

**Table 8:** Multiple Affymetrix *FKER* probe sets (transcripts) highly expressed in liver of E16 embryos

Affymetrix ID	SYMBOL	GENE NAME	Log2 FC	*GGA*	ACC. NUM.	REFSEQ ID
Gga.8960.3.S1_at	FKER	feather keratin 1-like	3.91	25	BX270499	NM_001277801
Gga.6482.1.S1_at	LOC431324	keratin A	3.43	25	BX262674	NM_001101732
Gga.8960.2.S1_at	FKER	feather keratin 1	3.18	25	AAA48930	NM_001081702
Gga.6264.1.S1_at	FKER	feather keratin 1	2.76	1	BI064513	NM_001277751
Gga.8960.1.S1_at	FKER	feather keratin 1-like	2.37	25	BX950836	NM_001277929
Gga.6558.1.S1_at	FKER	feather keratin 1-like	2.28	25	NM_001277960	NM_001277960
Gga.7285.1.S1_at	FKER	feather keratin 2-like	1.89	27	XM_424568	XM_424568

Six of these *FKER* transcripts were the highest DEGs found in liver of E16 vs. E18 embryos. GenBank Accession number BI064513 is our chicken abdominal fat cDNA clone pgf1n.pk001.j5, which was sequenced from a normalized chicken abdominal fat cDNA library

*Abbreviations*: feather keratin (*FKER*), fold-change (*FC*), *Gallus gallus* (*GGA*) chromosome, GenBank accession number (*ACC. NUM*.)

## Conclusions

The present analysis of the embryo-to-hatchling transition in meat-type chickens provides the first detailed view of the choreography of innate and dynamic transcriptional responses made by embryos and newly hatched chicks during the critical metabolic jump from lipid-laden ectotherm to free-living self-sufficient endotherm, respectively. The metabolism of late (E16-E20) embryos is dominated by β-oxidation of yolk lipids, glycolysis and gluconeogenesis, which utilizes the residual albumen and other proteins in allantoic fluid ingested by the embryo just prior to hatching. These metabolic processes are precisely controlled by multiple and interacting transcription factors (*PPARA, PPARGC1A, NR1H4* and *SIRT1, SERTAD2, KLF11, KLF13* and *KLF15*) and scores of metabolic enzymes, transporters, phosphatases and kinases. The metabolites derived from these processes interact with nuclear receptors, G-coupled protein receptors (GCPRs), transmembrane receptors and extracellular factors. Highly-expressed hepatic *DIO3* inactivates thyroid hormones in the embryo, while its expression sharply declines in hatchling chicks. Although hepatic expression of *DIO1* was rather constant during the peri-hatch period, another thyroid hormone-activating enzyme *DIO2* reached peak abundance at two ages, E20 and D9. Upon pipping through the egg shell, the hatching chick immediately converts to the pro-hormone T_4_ into metabolically-active T_3_, which supports achievement of thermodynamic freedom in hatchlings and synergetic activation of *THRSPA*, the major lipogenic transcription factor in chickens. Furthermore, T_3_ bound to its receptor (THRB) forms heterodimers with other nuclear receptors, which then control programmed expression of major lipogenic enzymes (*FASN, ME1, SCD, ELOV2, ELOV25, ELOV26, LSS*, *AGPAT2, ACACA, CYP7A, DHCR7* and *DHCR724*). In addition to *THRSPA*, other transcriptional regulators (*PPARG, PPARD, LPIN1, KLF11* and *SREBF2*) also interact and contribute to the sharp increase in lipogenesis observed in liver of D3 and D9 hatchlings.

Our present observation of the extraordinary expression of multiple *FKER* transcripts in liver of E16 embryos from meat-type chickens validates our original discovery of highly-expressed *FKER* gene in liver of e15 Leghorn (egg-type) chickens. We have proposed that hepatic expression of *FKER* in e15-e16 embryos could be an adaptive response to the coincident and massive accumulation of yolk lipids. This idea is supported by the exceptional expression of multiple β-keratin transcripts, including *FKER*, in the lactating crop sac of the pigeon [71], which accumulates and synthesizes lipids in production of “crop milk” that nourishes the altricial squabs. Many of the DEGs involved in de novo synthesis of triglycerides in the pigeon’s lactating crop sac are also found in liver of hatchling chicks, including the major lipogenic transcription factor *THRSPA*. Nevertheless, the biological function of *FKER* expressed in liver of e15-e16 embryos and the relationship between hepatic *FKER* and cutaneous *FKER* remain to be elucidated by definitive study. The present study provides new insight into dynamic interaction of multiple transcription factors and their direct target genes that provide *homeorhetric* regulation of metabolism during the abrupt embryo-to-hatchling transition of the domestic chicken, *Gallus gallus*.

## Additional files

Additional file 1: Experimental design of microarray hybridizations. A Microsoft Excel file containing a single work sheet “Array Hybridization Design” which describes the hybridization scheme used for the 24 Affymetrix Chicken Genome Arrays. (XLSX 12 kb)

Additional file 2: Primers used for qRT-PCR analysis. A Microsoft Excel file containing a single work sheet “qRT-PCR Primer Information”. This table provides the chicken gene symbol, GenBank ID number, 5′-3′ sequence for forward and reverse primers, and amplicon size (bp) for each gene used for qRT-PCR analysis. (XLSX 12 kb)

Additional file 3: Annotated gene list for 14 pairwise contrasts of liver transcriptomes in embryos and hatchlings across six ages during the peri-hatch period (E16-D9). The last worksheet provides the list of 1005 commonly-shared AR-DEGs identified by the Venn diagram shown in Fig. 3b. Each worksheet provides a list of the “Analysis Ready” (AR)-DEGs (FDR adjusted *P* ≤ 0.05; ±0.75 cutoff) for each contrast. (XLSX 905 kb)

Additional file 4: Canonical and biological pathways identified by the contrast of all embryos vs. all hatchlings. The AR-DEGs from the E vs. H contrast were assigned by Ingenuity Pathway Analysis (IPA) to canonical and functional and pathways. Fisher’s Exact Test is used in IPA to determine probability (*P* ≤ 0.05) that AR-DEGs belong to a particular canonical pathway or biological function accrued in the Ingenuity Knowledge Base. (XLSX 53 kb)

Additional file 5: Canonical and biological pathways identified by IPA in the E18 vs. E20 contrast. The AR-DEGs from the E18 vs. E20 contrast were assigned by IPA to overrepresented canonical and functional pathways. IPA uses Fisher’s Exact Test to determine probability (*P* ≤ 0.05) of each AR-DEG belonging to a particular canonical pathway or biological function. (XLSX 36 kb)

Additional file 6: Canonical-biological pathways identified by IPA in the contrast of E18 embryos vs. D3 hatchlings. The AR-DEGs identified in the E18 vs. D3 contrast were assigned by IPA to overrepresented canonical and functional and pathways. IPA uses Fisher’s Exact Test to determine probability (*P* ≤ 0.05) of each AR-DEG belonging to a particular canonical pathway or biological function. (XLSX 44 kb)

Additional file 7: Canonical and biological pathways found by IPA in the D1 vs. D9 hatchling contrast. The AR-DEGs identified in the D1 vs. D9 contrast were assigned by IPA to overrepresented canonical and functional and pathways. IPA uses Fisher’s Exact Test to determine probability (*P* ≤ 0.05) of each AR-DEG belonging to a particular canonical pathway or biological function. Each worksheet provides a list of AR-DEGs assigned to particular canonical pathways or biological functions, annotated with gene symbol, Entrez name, log2 ratio, cellular compartment, type and Entrez protein ID. (XLSX 31 kb)

Additional file 8: Pearson’s correlation analysis of gene expression levels of 15 “candidate” DEGs determined by genome microarray and verified by qRT-PCR analyses. A Microsoft Excel file containing the average normalized expression levels (log2 ratios) of 15 candidate DEGs as determined by genome microarray analysis and verified by qRT-PCR analysis. The Pearson’s correlation coefficient (*r* = 0.9287; *P* ≤ 0.01, 11 degrees of freedom) indicates a highly significant correlation between gene expression levels obtained from both microarray and qRT-PCR analyses. (XLSX 16 kb)

## Abbreviations

ANOVA: Analysis-of-variance
AR: Analysis Ready
aRNA: Amplified RNA
Ct: Average cycle time
DEGs: Differentially-expressed genes
E16: Embryonic day 16
FDR: False discovery rate
FKER: Feather keratin
GEO: Gene Expression Omnibus
*GGA*: *Gallus gallus* chromosome
GLM: General linear mode
IP: Ingenuity Pathway Analysis
LIMMA: Linear Models for Microarray Data
LSD: Least significant difference
MIAME: Minimum information about a microarray experiment
NCBI: National Center for Biotechnology Information
qRT-PCR: Quantitative reverse transcriptase polymerase chain reaction
RIN: RNA integrity number
SAS: Statistical Analysis System
TF: Transcription factor
THRSPA: Thyroid hormone responsive Spot 14 protein, alpha
